# Effects of Biotin on a Fluorescein‐Based Photosensitizer Revealed by Multiscale Computational Modeling

**DOI:** 10.1002/chem.71351

**Published:** 2026-07-06

**Authors:** Valeria Butera, Alessandra Gilda Ritacca, Giampaolo Barone, Nino Russo, Marta Erminia Alberto

**Affiliations:** ^1^ Department of Biological Chemical and Pharmaceutical Sciences and Technologies (STeBiCeF) University of Palermo Palermo Italy; ^2^ Department of Chemistry and Chemical Technologies University of Calabria Rende (CS) Italy

**Keywords:** DFT, MD, membrane, PDT, photochemistry

## Abstract

Biotinylation of Type II photosensitizers has recently been reported as a new efficient strategy to greatly boost the generation of both superoxide and singlet oxygen. However, the unexpected role of biotin in triggering Type I photoreactions, has not been yet established. We herein provide a thorough multiscale investigation of the effect of biotinylation on a fluorescein‐based photosensitizer (**1 **vs **1‐biotin**), investigating whether the two‐component conjugate is able to contribute to synergistic Type I/Type II photoreactivity. Membrane‐interaction studies show that the two agents exhibit a different behavior within the cell membrane despite both spontaneously permeate it even in the absence of biotin receptors. Remarkably, the presence of biotin promotes local accumulation of the dye inside the membrane not observed for the not‐functionalized photosensitizer, increasing local bioavailability. Our QM results show that the major effect on the ability to afford Type I‐PDT, comes from the different response to the typical acidic tumor microenvironment shown by the biotinylated *vs* not‐substituted compound. To the best of our knowledge, this is the first time that the positive effects of such functionalization have been linked with i) an increased membrane accumulation in absence of specific receptors, ii) a different acid‐base equilibrium influenced by its presence.

## Graphical abstract

Biotinylation of a fluorescein‐based photosensitizer enhances its performance in PDT through a synergistic dual action. In addition to Type II singlet oxygen generation common to both dyes, the biotinylated system induces stronger membrane perturbation and higher local accumulation in lipid membranes, associated with increased water permeability and altered membrane order and fluidity.

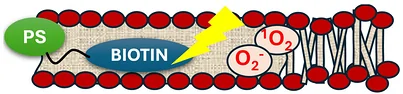

## 1 Introduction

Photodynamic therapy (PDT) has been explored as a non‐invasive, highly efficient cancer treatment strategy that destroys cancer cells using reactive oxygen species (ROS) generated from photosensitizers (PSs) and light of proper frequency [1, 2, 3, 4]. In this regard, anticancer PDT represents a valid alternative to conventional treatment methods, such as chemotherapy, surgery and radiotherapy [5], since it ensures a greater spatio‐temporal control resulting in a more selective and localized approach.

The generation of the highly reactive oxygen species occurs through two different mechanisms, which differ in the way the long‐lived lowest triplet excited state of the photosensitizer (T_1_) interacts with O_2_ [6]. In the Type I mechanism, T_1_ promotes redox reactions with biological substrates also including electron transfer to O_2_ to form the superoxide anion radical (O_2_^•−^). On the other hand, Type II pathway involves an energy transfer mechanism to O_2_, resulting in the formation of singlet oxygen species (^1^O_2_). Up to now, a variety of photosensitizers have been approved and successfully applied, most of which are involved in Type II mechanism [7, 8]. However, the efficacy of traditional Type II PDT is entirely contingent on the presence of sufficient oxygen in the tissues to generate cytotoxic singlet oxygen species. Unfortunately, some of the most aggressive and drug‐resistant cancers are accompanied by acute hypoxic conditions that hamper treatment outcome. Moreover, even with partial tissue oxygenation, the vascular collapse promoted by PDT, or the formation of ROS using high light fluence rates, can deplete itself the tumor oxygenation aggravating the hypoxia condition [9]. Type I PDT, on the other hand, offers an effective strategy to mitigate this issue due to its reduced oxygen‐dependence ensured by the participation of superoxide in the superoxide dismutase‐triggered catalytic cascades, which partially regenerates molecular oxygen. This enables a sustained ROS production even under hypoxic conditions [10]. At present, research into TypeI or dual TypeI/II PSs spans a diverse range of materials, from organic dyes [11, 12] to inorganic nanoparticles [13] and, especially, transition metal complexes [14, 15, 16, 17, 18, 19, 20, 21, 22, 23, 24, 25, 26, 27, 28], each offering distinct advantages in ROS generation at low oxygen levels.

Among the several approaches proposed so far, Song, Zheng and collaborators [29] have recently presented a novel strategy based on the incorporation of biotin unit on three organic photosensitizers, leveraging the well‐known preferential tumor‐targeting ability of biotin, through specific receptors that are overexpressed on cancer cells surface or tumor vasculature systems compared with normal tissues [30, 31, 32, 33]. Notably, they reported that the presence of biotin unexpectedly enhanced O_2_^−•^ generation of the selected PSs. Particularly, compared to **1**, the conjugate **1‐biotin** (see Scheme 1) was experimentally suggested to trigger the formation of superoxide anion radical and was chosen for the analysis of the PDT performance against tumor hypoxia.

**SCHEME 1:**
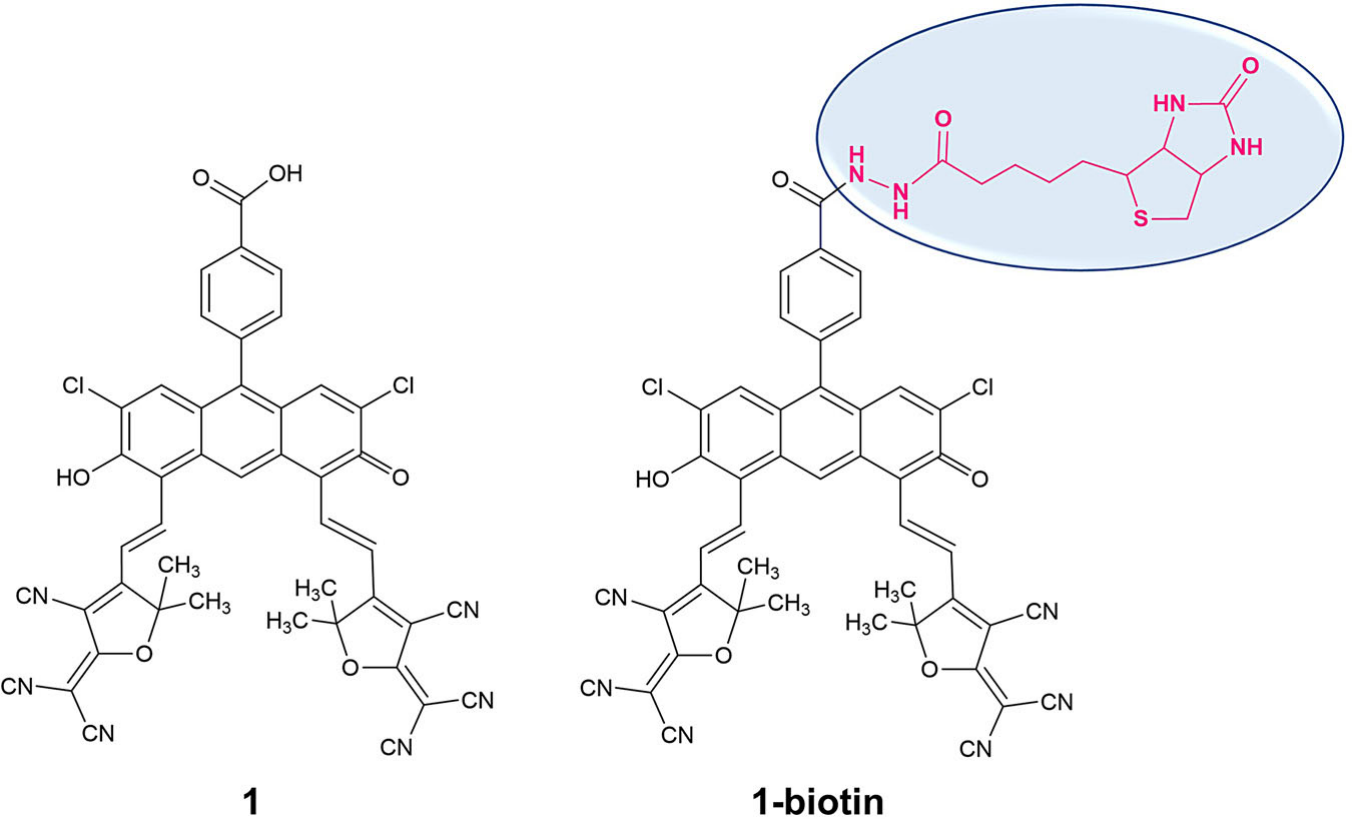
Investigated **1** and **1‐biotin** compounds.

To disclose the effective role of biotin in improving the ability of generating O_2_^−•^, we herein present a multiscale computational investigation based on a combination of classical Molecular Dynamics (MD), density functional theory (DFT) and its time‐dependent extension (TDDFT), to explore from one side, the impacts of both species on the structural properties of cell membranes and, from the other side, the photophysical behaviors of the photosensitizer upon biotinylation.

Membrane‐interaction studies show that both PSs spontaneously enter in a pure POPC membrane, by means of a passive diffusion mechanism despite the lack of biotin‐receptors, and they can easily accumulate inside the cell membrane, with biotin promoting an increased local bioavailability compared to **1**. Moreover, a stronger membrane perturbation was observed for **1‐biotin** caused by the decrease of the thickness and the increase of the area per lipid.

Our DFT/TDDFT results show that the primary factor influencing the major ability of **1‐biotin** to act through Type I, is the different response to the intracellular acidic tumor microenvironment observed, compared to **1**. Indeed, following the initial study of the photophysical behavior of the neutral **1** vs **1‐biotin**, our exploration has been extended in order to take into account different protonation states of them, considering the pH‐dependence of such photosensitizers in the common acidic environment of the tumor cells (typically around pH 5.3) [34].

Our results have pointed out that in the range of 4<pH<6.4, the mono‐anionic form of **1** (**1^−^_c_**) and the neutral **1‐biotin** are the predominant ones and, more importantly, they reveal that the photogeneration of O_2_^−•^ is more likely to occur if triggered by the biotin‐containing species.

As far as we know, this is the first time that the positive effects of such functionalization have been linked to newly proposed effects, beside the well‐known selective accumulation. Indeed, our data show that biotin is able to promote an interesting synergy between i) higher membrane perturbation enabling ROS to deeply permeate within cells causing an increased oxidative damage and ii) different acid‐base equilibria influenced by its presence.

## 2 Results and Discussion

### 2.1 MD Simulations of Membrane Insertion Mechanism and Perturbation Effects

As a first step of our investigation, we focused on the study of the insertion mechanism of compound **1** and its biotinylated derivative **1‐biotin** into the cell membrane to analyze any differences or similar behaviors in the internalization process, between the two compounds. From the experimental point of view, indeed, it is quite accepted that biotin‐conjugated compounds enhance the drug uptake by cancer cells, [30, 31, 32, 33, 34, 35] exploiting their overexpressed biotin receptors. Mitochondria and/or lysosomes are usually the sub‐cellular organelles in which they accumulate [34]. Anyway, no data are available on the effect of such substituent on the insertion in cell membranes where receptors are less expressed. To add some insights on this aspect, we explored the insertion mechanism of both molecules within the membrane bilayer, by means of MD simulations. The initial model systems including **1** and **1‐biotin** immersed in a slab of water and POPC membrane bilayer are shown in Figure S1. Our simulations show that the insertion within the membrane bilayer spontaneously occurs for both **1** and **1‐biotin**, within 1µs of trajectories, reported in Figure 1a,b respectively, initially approaching through the fluorescein moiety (Time I, II, Figure 1). The conjugated biotin, once entered, is kept at the level of the phospholipid heads, interacting with phosphate groups at Time III (Figure 1b) allowing the fluorescein moiety to penetrate more deeply inside the membrane.

**FIGURE 1:**
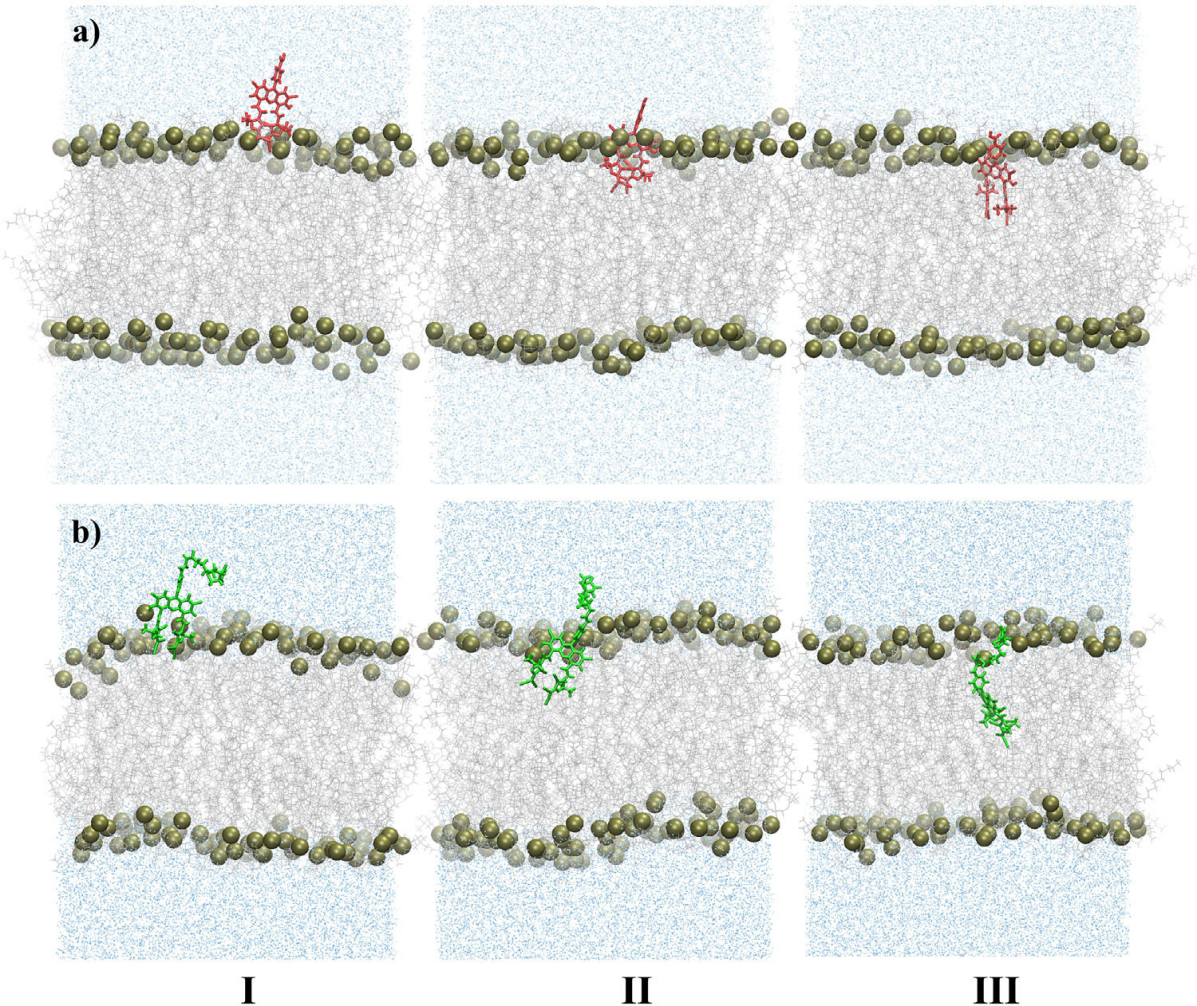
Snapshots extracted from MD simulations at consecutive time frames (I–III) showing the spontaneous insertion of (a**) 1** and (b**) 1‐biotin** into the membrane (POPC lipid model), occurring within 1 µs, respectively. **1** and **1‐biotin** molecules are shown in licorice in red and green, respectively. POPC lipid phosphorus atoms are shown as tan spheres and acyl chains as grey lines. Water molecules are reported in light blue dots.

A higher propensity to interact with the hydrocarbon tails, enabling access to the membrane's hydrophobic core, has been linked to the increased cytotoxicity of previously explored drugs [36]. Both compounds remain localized within the membrane bilayer for the entire simulation time (1 µs), without crossing from a leaflet to another, as shown in Figure 2a. This behavior was confirmed through a set of three independent MD replica simulations, as reported in Figure S2. The spontaneous insertion by passive diffusion mechanism verified by our calculations, no matter whether the presence or not of biotin‐receptors, agrees with what was previously experimentally ascertained also for an analogue xanthene‐based photosensitizer which entered the membrane, specifically localizing in the lysosomes, even in absence of specific receptors [37].

**FIGURE 2:**
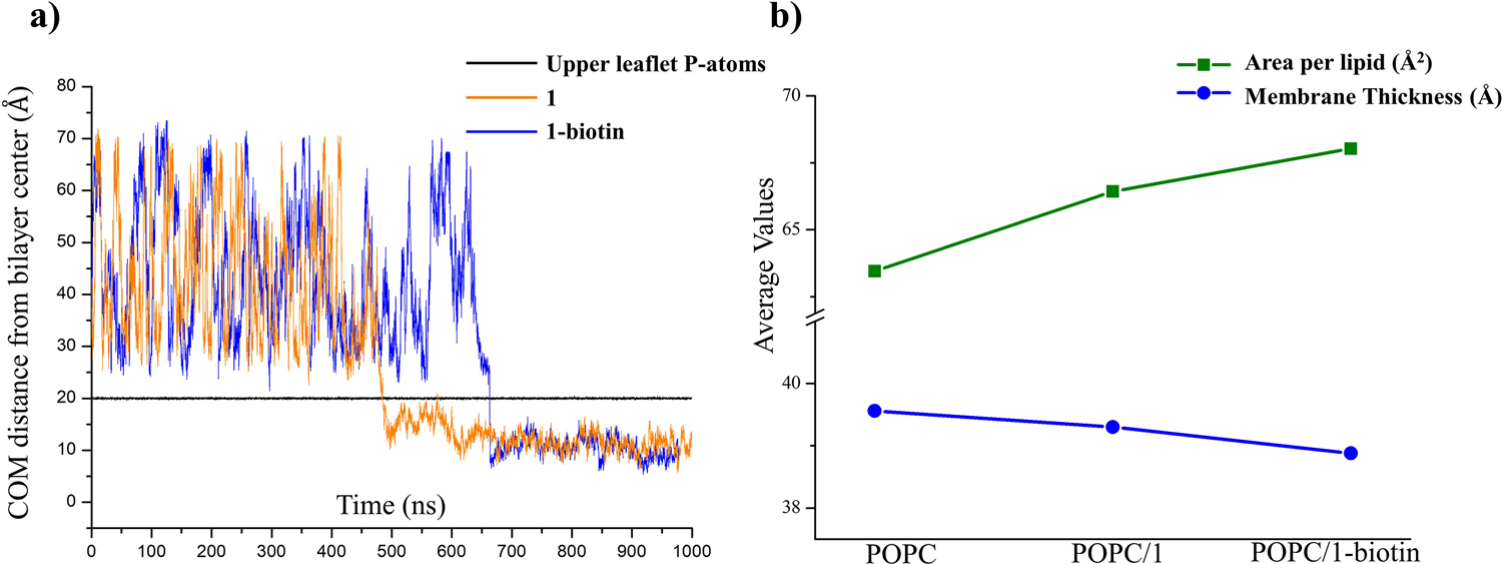
(a) Time‐dependence of the distance between the center of mass (COM) of **1** and **1‐biotin** and the bilayer center, in the two different MD trajectories; the black line is the average position of the upper leaflet lipid head groups (P‐atoms); (b) Average values of area per lipid (green) and thickness (blu) membrane parameters without any embedded molecule (POPC), with ten embedded molecules of **1** (POPC/1) and ten of **1‐biotin** (POPC/1‐biotin).

Accordingly, our findings suggest that both PDT potential agents can be internalized in the membrane regardless of biotin's presence, still being obvious that the presence of specific receptors could enhance the drug targeting. To gain deeper insights into the process occurring inside the membrane, also providing data on potential membrane alterations induced by the presence or absence of the biotin, we performed two simulations of 1µs each of already embedded species, five per leaflet, in the ground state, as shown in Figure S3a,b. Even in this case, both **1** and **1‐biotin** compounds stably dwelled inside the membrane for the whole simulation time. The analysis of the crucial membrane structure parameters in the presence and in the absence of **1** and **1‐biotin** reported in Figures 2b and S4 (Upper panel), reveals that once the dye permeates the cells’ membrane it induces a slight shrinkage of membrane thickness and an increase of area per lipid, compared with the POPC membrane alone. While the addition of **1** modifies only the area per lipid with a slight difference in membrane thickness, **1‐biotin** induces a stronger membrane perturbation decreasing the thickness and increasing the area per lipid, in a more significant amount. Such structural modifications can be connected with an increase of the water permeability and, in general, with alterations in the lipid membrane order and fluidity, [38] ultimately allowing reactive species, such as ROS, to enter the cell and cause oxidative damage to intracellular macromolecules, such as DNA or proteins. Similar changes in the leaflets’ distance have been previously connected with a variation of membrane potential that can cause a disruption of membrane functions resulting in cell death [39].

Furthermore, it has been observed a higher tendency of **1‐biotin** over the not‐substituted **1** to locally accumulate by interacting with neighboring compounds, ultimately forming clusters (Figure 3a–f).

**FIGURE 3:**
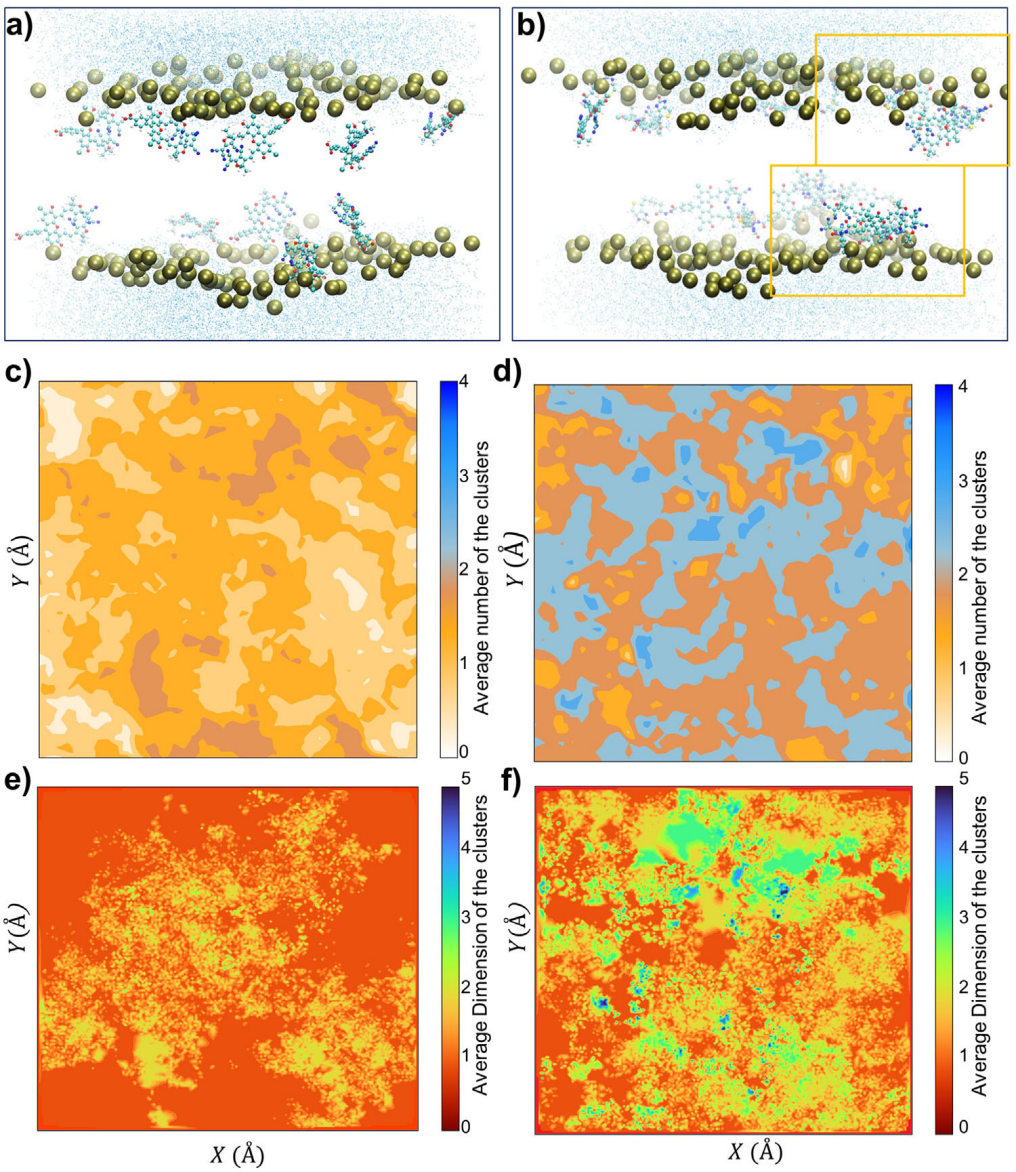
Representative snapshots extracted from MD simulations showing (a) the random distribution of **1** dye and (b) the local clusters generated by **1‐biotin;** (c) Average number of clusters (from 0 to 4) formed during 1 µs of MD simulation of **1** and (d) **1‐biotin** dyes embedded in the POPC membrane. Color scale progresses from white (0) to warm beige‐orange (1–2) to light blue to blue shades (3–4), indicates increasing probability of aggregation. X and Y axis (in Å) represent the spatial coordinates of the membrane system plane. Panels (e) and (f) report the average dimension of clusters (within a cut‐off = 4 Å) forming in **1** and **1‐biotin** MD simulations, respectively. X and Y (in Å) represent the coordinates of the center of mass of the molecules in the membrane x‐y plane during the simulations. Color gradient from red to light and dark blue indicates a higher number of interacting molecules within 4 Å, from zero (red) to more than three interacting molecules (green to blue).

Representative snapshots extracted from MD simulations, showing the absence and the formation of aggregates, are reported in Figure 3a,b for **1** and **1‐biotin**, respectively. A thorough analysis of the two trajectories quantifying the number of aggregates spontaneously forming during the simulation time clearly shows that biotin‐substituent promotes the formation of a higher number of clusters, from 2 to 4 compared with **1**, as can be observed in the 2D spatially resolved maps (Figures 3c,d). Regions of the maps with lower aggregate density, highlighted in warm beige‐orange, are predominant in the analysis of the trajectories obtained for compound **1**. In contrast, areas with a higher number of clusters, marked in light blue to blue shades in the 2D map, distinctly characterize the simulation behavior of the biotinylated compounds (**1‐biotin**), indicative of a more pronounced tendency to form aggregates. To further investigate this interesting tendency toward a selective accumulation observed for **1‐biotin**, we examined the average dimension of clusters detected in both simulations quantifying the number of **1** and **1‐biotin** involved in the formation of each aggregate (Figures 3e,f). When the biotin substituent is lacking (Figure 3e), the few clusters found have maximum dimension of 2 (rust regions of the map), while, data obtained for **1‐biotin** (Figure 3f), show higher dimensions for the aggregates which include 3, 4 and 5 molecules of **1‐biotin** locally interacting, as revealed by colors orange to blue, respectively. A structural analysis involving the radial distribution function (RDF) and hydrogen bond interactions analysis (see Figures S5, S6, respectively), between **1‐biotin** dyes in the clusters suggested us that the driving force for the aggregation is mainly due to presence of intermolecular H‐bonding involving mostly biotin moieties. Indeed, while the g(r) indicates that the probability of finding all atoms of the species within the cut‐off distance (4Å) is higher for **1‐biotin** than for **1** (see red dotted lines in Figure S5a), this trend is reversed when calculating the g(r) just considering individual fragments without including H atoms, thus restricting the analysis to fluorescein components and biotin moiety within the cut‐off distance (Figure S5b). This suggests a greater involvement of hydrogen atoms in the formation of aggregates in **1‐biotin**, as also confirmed by the higher density of persistent hydrogen bonds shown in Figure S6. Moreover, a visual inspection of the aggregates structures with different dimensions and reported in Figure S7, shows how some persistent H‐bond interactions extracted by the H‐bond analysis, biotin‐biotin and biotin‐fluorescein types, can effectively contribute to their structural stability. On the basis of such outcomes, it is possible to hypothesize that the local accumulation of the dye inside the membrane is due to the presence of biotin.

A different POPC‐membrane composition containing ca. 11% of cholesterol (CHL) [38] was also used to verify the structural perturbations induced by the presence of **1** and **1‐biotin**. Cholesterol plays an important structural role in the cell membrane since it is responsible for lipid order and rigidity. Indeed, it is known that plasma membranes of cancer cells have lower cholesterol levels especially for metastatic cells of breast cancer [40, 41, 42, 43]. A decrease of its concentration leads to an increase in fluidity and permeability, making the membrane more vulnerable to ROS attacks. Thus, we modelled this condition by including 20 cholesterol molecules in the systems with and without **1** and **1‐biotin** (Figure S3c,d). The calculated area per lipid and the thickness of the bilayer for the POPC/CHL, POPC/CHL/1 and POPC/CHL/1‐biotin mixtures, are reported in Figure S4 (Lower panel) and Table S1. Compared to the systems without CHL (Figure 2b), the addition of cholesterol induces an increase of the stiffening effects on the lipids and a higher order of the membrane resulting in a decrease of the overall area per lipid and an increase of the bilayer thickness. Although the POPC/CHL mixture presents a tightly packed structure which tends to protect the membrane, the presence of **1** and **1‐biotin** still modulates in a small amount its properties. The perturbative effect induced by **1‐biotin** is more evident in the reduction of the membrane thickness, while the increase of area per lipid occurs with both **1** and **1‐biotin** with no significant differences (Table S1).

### 2.2 DFT and TDDFT Exploration of Photophysical Properties of **1** and **1‐Biotin**

After confirming the membrane permeation and retention of both dyes, with more pronounced alterations and local accumulation observed in the presence of biotin, we investigated how this functionalization affects their photophysical properties. To this end, we explored both **1** and **1‐biotin** species to determine whether biotin exerts any modulating effect. Given the acidic pH of the tumor microenvironment (typically pH 5.3), one cannot help but consider the acid–base equilibria in which both compounds **1** and **1‐biotin** might be involved, once the membrane has been crossed. Indeed, both compounds bear an acid group on the xanthene core that could be deprotonated. In addition, **1** also has a carboxylate moiety, which is functionalized through biotinylation. As a consequence, while compound **1** could afford two different mono‐anions, one on the xanthene core (**1^−^**) and one leading to the carboxylate species (**1^−^c**), for **1‐biotin** the deprotonation could occur only on the xanthene core (**1‐biotin^−^**). It is thus paramount to consider the pH‐induced changes in the corresponding molecular structures and, therefore, how those changes might influence the photoactivity. The pH dependence of the various species of fluorescein in aqueous solution has been widely investigated in the past, [40, 41, 42] and it is well known that the relative acidities of the benzoic and xanthene acid groups differ by two orders of magnitude (p*K*a = 4,00 and 6,4, respectively) [44, 45, 46], so that the deprotonation process leading to the **1^−^c** species (see Scheme 2a) is the only possible one at the considered pH.

**SCHEME 2:**
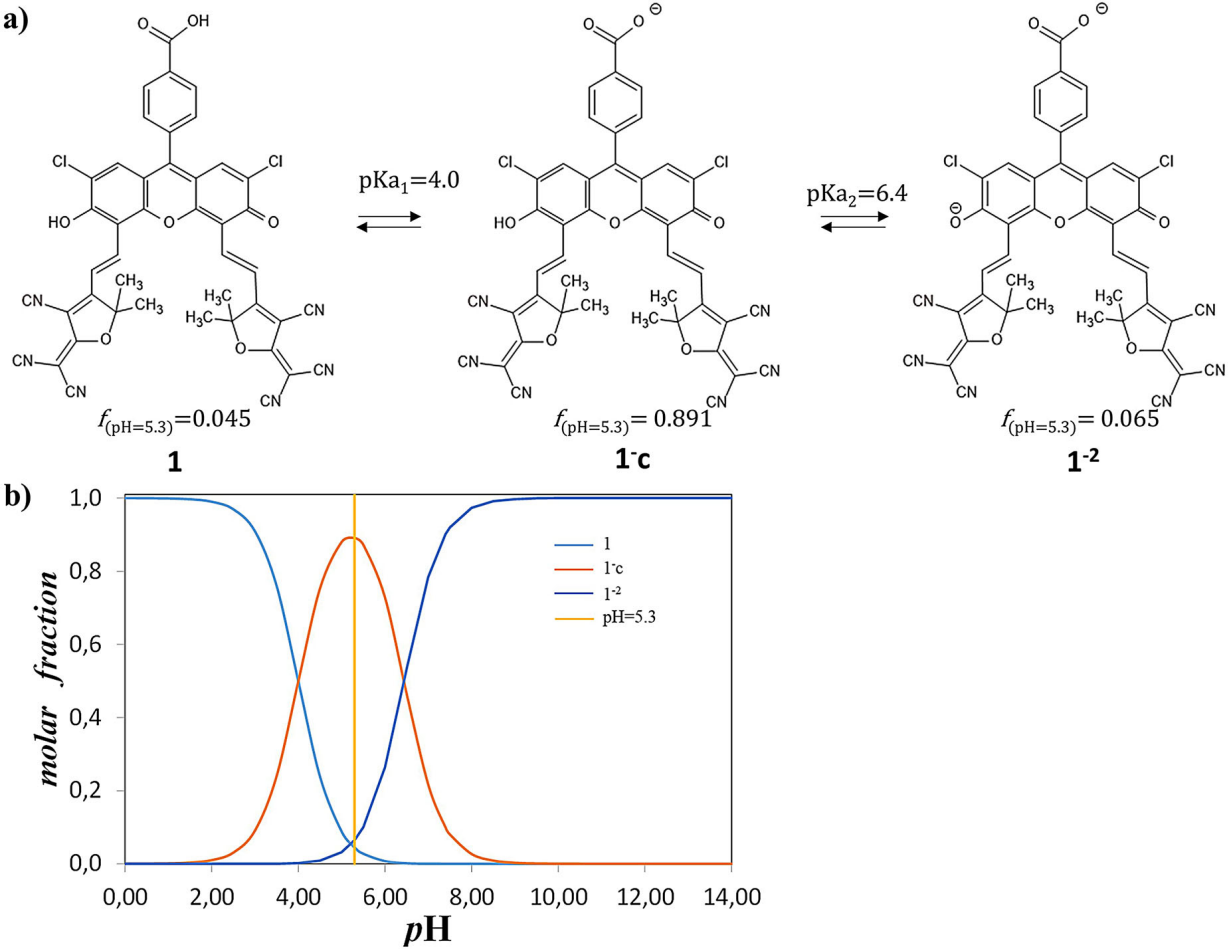
(a) Considered neutral and deprotonated species and their calculated $pK_{a}$ values; (b) pH‐dependent distribution curve showing the relative abundance of the various protonation states of the compounds.

To further explore this acid‐base equilibrium, we adopted the parameter fitting method previously proposed [47] confirming the reported p*K*a for the deprotonation of benzoic acid group and providing the pH‐dependent distribution curve reported in Scheme 2b. The curves were obtained by using the molar fractions of the neutral, monoanionic and dianionic species at the considered pH = 5.3, as devised by applying the aforementioned procedure [47]. Additional computational details are reported in the Supporting Information. They clearly show the relative abundance of the various protonation states of compound **1** and they clearly indicate that at the slightly acidic pH, the monoanionic species **1^−^c** is the predominant form, being 89:4 the relative abundance (%) of mono‐anion **1^−^c** vs **1**, respectively.

The **1^−2^** species contributes to a much lower extent (8%). As for the biotinylated compound, in tumor microenvironments the neutral form is expected to prevail due to the absence of benzoic acid group.

The same hypothesis could be done even in a lipidic environment. An increase in p*K*a is generally expected in a lipid environment due to the reduced dielectric constant and the limited stabilization of charged species. In this context, although the strong dependence of p*K*a on the local microenvironment is straightforward to establish, the greater exposure of the carboxylic group to interfacial water, as suggested by our MD simulations, may still allow deprotonation at acidic pH, even in the presence of a p*K*a shift. Conversely, the xanthene moiety, being more deeply embedded in the lipid phase, is expected to experience a more pronounced p*K*a increase, which would further disfavor the formation of its anionic form and is therefore consistent with the absence of the anionic biotin species already suggested in water.

As a consequence, the **1^−^**, the **1‐biotin^−^** and the dianion species **1^−2^** could be all neglected at the considered pH.

Accordingly, the species primarily involved in the PDT process could be the monoanion **1^−^c** and the neutral **1‐biotin**, which will be the focus of the following discussion. From a structural point of view, as shown in Figure 4 and in agreement with the structure reported by An and collaborators [29], the biotin chain (pink fragment) is folded above the xanthene core (green fragment), while the hydrofuran terminals (gray fragments) lie on two planes, almost parallel to each other, with the oxygen atoms pointing towards the same direction. From a photophysical point of view, neither biotin nor its folded conformation have a significant impact on the absorption properties of the molecule. Indeed, in agreement with the similarity between fluorescein‐like compounds and analogues containing different groups in place of the carboxyl group already previously highlighted [46], and also in line with the experimental data previously reported [29], **1** and **1‐biotin** display almost superimposable UV–vis spectra in both water and cyclohexane solvent (Figure S8). Consistent with the experiments, the lowest energy transitions centered at 461 and 476 nm for **1** and **1‐biotin**, respectively, are compatible with that around 490 nm reported experimentally. Inspection of the NTO involved in the S1 bright transition of **1‐biotin** shows only a minimal contribution of the sulfur atom (Figure 4d), thus only partially supporting the hypothesis of An and collaborators [29], which indicates that the folded structure is expected to enhance effective electron transfer between biotin and xanthene cores. Frontier Molecular Orbital plots are also reported in Figure S9.

**FIGURE 4:**
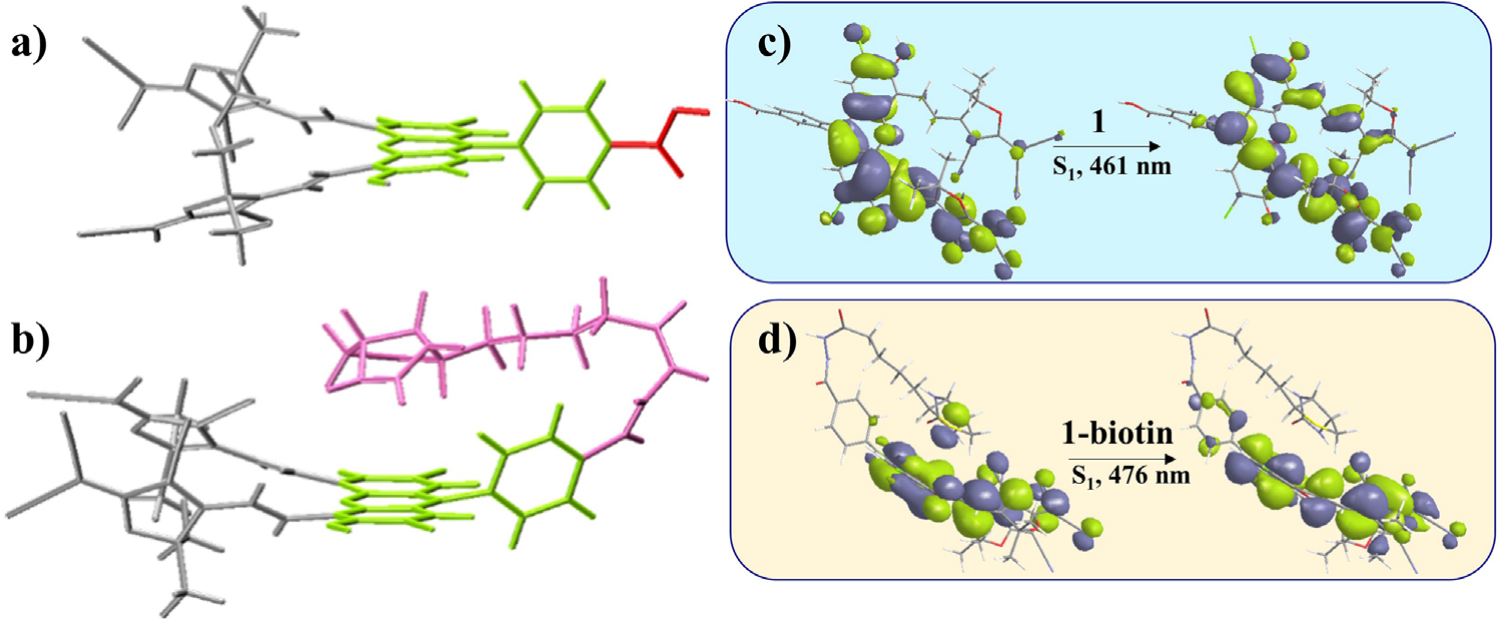
(a,b) Optimized structures of **1** and **1‐biotin** molecules at B3LYP/6‐31+G(d,p) in water environment; (c,d) Natural Transition Orbitals (NTOs) describing the lowest energy singlet‐singlet transition (S1), at the TDDFT/CAM‐B3LYP//6‐31+G(d,p) level of theory, in water environment.

The less intense shoulder observed experimentally at *λ*>600 nm [29] is consistent with a mixture of deprotonated species in the sample, in particular the dianionic **1^−2^** species, whose computed spectrum shows a red‐shifted band at 530 nm in addition to the main one around 450 nm (Figure S8b).

Indeed, while the presence and state of ionization of the carboxyl group does not induce major effects on the absorption properties of the molecule, as can be further confirmed by comparing the **1^−^c** and the neutral species **1** (See Figure S8b), the spectra calculated for the other anionic species (**1^−^** and **1^2−^ and 1‐biotin^−^**) have a notable impact on the absorption behaviors (Figure S8b,c). The main singlet‐singlet transitions along with their energy, intensity and natural transition orbital description (NTO) for all the protonation states, are reported in Table S2 and Figure S10.

### 2.3 Type I and II Mechanisms

The feasibility of *Type II* photoreactions relies on an efficient formation of the singlet cytotoxic species ^1^O_2_ (generally referred as singlet oxygen quantum yield, *φ*_Δ_) that occurs by an energy transfer from the excited triplet PS to ^3^O_2._ For this process to happen, the first requirement is an efficient triplet state population of the PS that can only be achieved by intersystem crossing (ISC) process from a singlet state. Moreover, the singlet−triplet energy gap of the PS must be larger than that of the O_2_, thus promoting the ^3^Σ_g_^−^ → ^1^Δ_g_ transition. The computed Δ_S‐T_ for oxygen is 0.94 eV, in good agreement with the experimental value of 0.98 eV [48, 49]. Three triplet states lie below **S1** for all the considered species with a proper energy to promote the formation of the singlet oxygen state, especially relevant for the most abundant **1^−^c** and **1‐biotin**. The three triplet states have energies comprised between 1.91 and 2.12 eV for **1^−^c** and between 1.89 and 2.22 eV for **1‐biotin**, in water, while maintaining similar results even in cyclohexane (Figure S11). Therefore, they all could well contribute to populate the T_1_ state by internal conversion (IC), provided that an efficient ISC process takes place from the excited singlet states. To establish the feasibility of the ISC process, SOC values between the first excited singlet and the underneath triplet states have been computed for the abundant **1^−^c** and **1‐biotin** species, and their values are reported in Table S3 of the Supporting Information, both in water and in a less polar environment (cyclohexane). As expected for organic molecules, SOC values are relatively small but still consistent with an efficient population of triplet states, as also previously found for the clinically approved Foscan molecule [50, 51, 52]. Besides the ability to produce singlet oxygen, the biotin functionalization has been suggested to enhance PDT efficacy under hypoxic conditions [29]. Consequently, we also investigated the photogeneration of ROS exploring the feasibility of *Type I* electron‐transfer reactions between the most abundant **1^−^c** and **1‐biotin** species and molecular oxygen, leading to the generation of highly reactive superoxide species.

To assess the possibility that highly reactive superoxide species could be formed by tested compounds, vertical electron affinity (VEA) and vertical ionization potential (VIP) of the relevant species were computed and used to establish the thermodynamics of the involved reactions (Table 1). Three long range separated XC functionals (Cam‐B3LYP, M11 and wB97XD) have been employed in light of their improved description of long‐range charge‐transfer interactions and their reduced self‐interaction errors. They are particularly well suited for systems involving ionic species, where accurate evaluation of vertical ionization potentials (VIP) and electron affinities (VEA), as well as reaction thermodynamics involving changes in charge distribution, is required. Table 1 reports the thermodynamic of the Type I PDT reactions obtained with the three XC functionals, while VEA and VIP can be found in the Table S4 of the Supporting Information. Looking at the computed values, all the tested XC functionals unequivocally indicate that both species can undergo reduction in their triplet states via autoionization, being predicted exothermic the reaction 2 for both compounds (Table 1 and Table S4). They also agree in confirming a lower electron‐accepting character (VEA) for **1 biotin** versus **1**, suggesting a more plausible competition between oxygen and biotinylated compound for electron transfer process (See Table S4). Indeed, analysis of reaction 3 suggests that the electron transfer to O_2_ could be more plausible for the biotinylated compound if compared with 1, in line with its reduced electron‐withdrawing character across all tested functionals. On the contrary, the more positive value of Δ*E* calculated for the same process in presence of **1^−^c**, underlines that the formation of superoxide is slightly disfavored by this species. Nonetheless, the slight difference between the calculated Δ*E* values for the two compounds prevents a definitive conclusion on this aspect, leaving open the question of whether the higher production of superoxide observed experimentally is effectively mediated by oxygen and boosted by biotin or whether other biological pathways are involved.

**TABLE 1:** Thermodynamics [Δ*E* = *E*(products) −*E*(reactants)] of the Type I PDT reactions based on the VEA and VIP values shown in Table S3, calculated at Cam‐B3LYP, M11, wB97XD/6‐31+G(d,p) level of theory. All energies are in eV.

	Type I photoreactions	Cam‐B3LYP		M11		wB97XD	
		**1^−^c**	**1‐biotin**	**1^−^c**	**1‐biotin**	**1^−^c**	**1‐biotin**
	**Autoionization reactions**						
(1)	^3^Ps + ^1^Ps → ^•^Ps^+^ + ^•^Ps^−^	0.30	1.01	0.42	0.63	0.34	0.56
(2)	^3^Ps + ^3^Ps → ^•^Ps^+^ + ^•^Ps^−^	−1.61	−0.88	−1.72	−1.44	−1.73	−1.49
	**Indirect reaction**						
(3)	^•^Ps^−^+ ^3^O_2_ → ^1^Ps + ^•^O_2_^−^	0.28	−0.07	0.09	−0.03	0.24	0.12
	**Direct electron transfer**						
(4)	^1^Ps + ^3^O_2_ → ^•^Ps^+^ + ^•^O_2_^−^	2.50	2.84	2.65	2.67	2.65	2.72
(5)	^3^Ps + ^3^O_2_ → ^•^Ps^+^ + ^•^O_2_^−^	0.59	0.95	0.51	0.60	0.58	0.67

It should be emphasized that neither the less abundant **1** species is able to promote superoxide formation via reaction 3, while the less prevalent anionic form of the functionalized compound (**1‐biotin^−^**) also shows a favorable process for indirect electron transfer through reaction 3. It is noteworthy that, although in smaller amounts, superoxide production was experimentally observed even in the presence of compound **1** [29]. This can be rationalized by considering the presence of the dianionic species of **1**, which contributes only marginally at the acidic pH considered in our study, while it is present in significantly higher amounts at the neutral pH used in the experimental setup. As a matter of fact, our data indicates that the indirect process (reaction 3) can also be promoted by the dianionic form (**1^2^^−^**) (Table S5). However, since acidic conditions preclude its presence, the generation of superoxide promoted by **1** should be even less in those conditions. As a consequence, the biotinylation is expected to play an even more crucial role in enhancing superoxide production and the promotion of the Type I mechanism, especially considering its higher intracellular accumulation that could improve their bioavailability in cancer cells.

### 2.4 Computational Details

#### 2.4.1 Ground and Excited States Properties (DFT/TDDFT)

All the calculations have been performed at DFT and its time‐dependent formulation TDDFT, including the Tamm–Dancoff approximation (TDA‐TDDFT) [53], with the Gaussian 16 software [54].

Ground‐state (GS) geometry optimizations of both neutral and anionic species were carried out at the B3LYP [55] level of theory and the 6–31+G** basis set was used for all atoms. On the other hand, all TDDFT calculations were performed using the most suitable CAM‐B3LYP functional as it provides an improved description of long‐range charge‐transfer excitations [56]. Water solvent environments were simulated by means of polarizable continuum model (PCM) [57] as implemented in Gaussian 16 for both GS optimizations and TDDFT calculations. Singlet–triplet energy gaps (Δ_S‐T_) were computed at the same level of theory used in TDDFT calculations, by exploiting the Tamm–Dancoff approximation [53]. The same protocol has been also used to determine the singlet‐triplet splitting gap of O_2_ by using the method proposed by Ovchinnikov and Labanowski [58] for correcting the mixed spin energies and removing the foreign spin components. This method has been already demonstrated to give very good agreement with the experimental values at different level of theory [48, 59]. Spin−orbit matrix elements were computed by using the TDDFT approach, as implemented in the Orca package [60, 61] at the first excited singlet state S_1_, using the mean‐field spin–orbit operator. The B3LYP functional and the relativistic ZORA‐DEF2‐SVP basis sets were employed [62]. The RIJCOSX approximations was also introduced to speed up the calculations’ time.

Spin−orbit couplings (SOCs) have been defined according to the following formula:

$$\mathrm{SO}{C_{i}}_{j}=\sqrt{\left|\psi_{S_{i}}\left|{\hat{\;H}}_{\mathrm{SO}}\right|{\left.\psi_{T_{j,n}}\right|}^{2}\right.}n=x,y,z$$

The p*K*a values have been estimated by solving the equation:

$$pKa_{\mathrm{calc}}=m\Delta Gs_{\left(\mathrm{BA}\right)}+C_{0}$$

where *m* and *C*_0_ are calculated according to the parameter fitting method previously proposed [47]. Then, the molar fractions of the neutral, anionic and di‐anionic species, present in aqueous solution, have been evaluated at pH = 5.3, according to the computational methodology suggested by Galano et al. [47]. (See Supporting Information for further details).

#### 2.4.2 MD Simulations

Membrane permeability of the two species (**1** and **1‐biotin**) were explored simulating their insertion in a pure 1‐palmitoyl‐2‐oleoylphosphatidylcholine (POPC) membrane immersed in a water box of 70 × 70 × 140 Å, by performing MD simulations. Three independent trajectories of 1 µs each have been run for every system, with one molecule of each complex initially located in the water phase without constraints (Figure S1). To provide insights into the different anticancer efficacy experimentally proposed for **1** and **1‐biotin**, their possible effects on the structural properties of the membrane have been explored by performing two simulations of 1 µs each, involving ten molecules of the two species (five for leaflet) within a bilayer of 188 POPC lipids (Figure S3a,b). A small‐scale comparative MD run of 250 ns each of POPC/Cholesterol (≈ 9:1 ratio), including 168 POPC lipids and 20 cholesterol molecules (ten per leaflet), have been performed to evaluate the perturbation effect of 1 and 1‐biotin in a more packed membrane structure (Figure S3c,d).

CHARMM‐GUI web server [63] was used to generate the initial coordinates of the systems using pre‐equilibrated lipid structures. The introduction of physiological amounts of counterions ensured the neutralization of the electrostatic charge of the system. All simulations have been run with NAMD v3.0 code [64] using the Amber force field [65] and the TIP3P model for water molecules. Parametrization of **1** and **1‐biotin** has been carried out starting from the B3LYP/ 6–31+G** atomic charges and by using easyPARM tool [66]. The time step for integrating the equations of motion was 2 fs, and covalent bonds to hydrogens were constrained using SHAKE algorithm [67]. Simulations were performed with periodic boundary conditions in the NPT ensemble, with p = 1 atm and T = 310 K, using a Langevin thermostat with a damping constant of 1 ps^−1^ and a Langevin piston [68] with a constant decay of 50 ps^−1^ and an oscillation period of 50 fs. A flexible unit cell was used with a constant ratio in the x−y plane. Long‐range electrostatic interactions have been computed using the particle‐mesh Ewald method [69], with a sixth‐order spline and 1 Å grid spacing. The analysis of the trajectories was conducted employing MDAnalysis tool [70, 71] and VMD software [72].

## 3 Conclusions

The biotinylation of a fluorescein‐based photosensitizer has been recently proposed as a strategy to boost the generation of both superoxide anion radicals and singlet oxygen, improving the efficacy of PDT. To shed light on the role of such substituent, we have adopted a multiscale computational approach combining membrane‐interaction studies at MD level and a photophysical exploration by means of DFT/TDDFT methods. The outcomes of the present investigation reveal that biotin fosters a synergistic combination of multiple mechanisms, leading to enhanced multimodal activity.

From one side, 1 µs‐long MD simulations demonstrate that both **1** and **1‐biotin** spontaneously permeate the membrane by means of a passive diffusion mechanism, even in the absence of biotin receptors. Notably, the analysis of the distinctive membrane structure parameters with **1‐biotin** molecules embedded into the membrane, highlighted a stronger membrane perturbation compared to dye **1**. Indeed, a significant shrinkage of membrane thickness and a concomitant increasing of the area per lipid was observed in the presence of the biotin‐functionalized agents, compared with the properties of a pure POPC membrane alone. Such structural modifications could be connected with an increase of the water permeability and, in general, with alterations in the lipid membrane order and fluidity. Moreover, the presence of biotin was found to promote local accumulation of the dye inside the membrane not observed for the not‐functionalized photosensitizer. Our data confirm that such aggregation is favoured by biotin.

From the other side, a thorough DFT/TDDFT exploration of the photophysical properties of **1** and **1‐biotin** provided insights on the TypeI and TypeII activity of the PS upon biotinylation, including the acid‐base equilibria in the typical acidic tumor microenvironment. Results confirm that both dyes are able to generate singlet oxygen species through the common energy transfer mechanism (TypeII). Analysis of Type I photoreactions shows that the most abundant species at low pH exhibit slightly different abilities to produce superoxide. The lower electron‐accepting character of the biotinylated compound may be consistent with oxygen competition for the electron transfer process. The oxygen reduction by the reduced form of the 1‐biotin appears somewhat more feasible. Anyway, the slight difference between the calculated ΔE values for this process promoted by the two compounds, leaves open the question of whether the higher production of superoxide observed experimentally is effectively mediated by oxygen and boosted by biotin or whether other biological pathways are involved

## Author Contributions

**V.B**. and **A.G.R**. contributed equally and share the first authorship. They carried out investigation, methodology, formal analysis and first draft writing. **G.B**., **N.R**., and **M.E.A**. data curation, writing – review and editing, resources. **M.E.A**. supervision, conceptualization, funding acquisition and project administration.

## Conflicts of Interest

The authors declare no conflicts of interest.

## Supporting information



Supplementary Information available: [MD Starting coordinates, MD Trajectories of spontaneous insertion, Membrane Thickness (Å) and Area per lipid (Å^2^) of 1 and 1‐biotin within POPC membrane; DFT Optimized structures, TDDFT Computed UV‐Vis spectra, Natural Transition Orbitals (NTOs), Molecular Orbital's plots, Spin‐orbit coupling matrix values (SOC), Thermodynamics of the Type I PDT reactions, of 1 and 1‐biotin molecules]. See DOI: 10.1039/x0xx00000x

## Data Availability

The data that support the findings of this study are available in the supplementary material of this article
