# Supplementary material for: Effects of Biotin on a Fluorescein‐Based Photosensitizer Revealed by Multiscale Computational Modeling
Source: Chemistry. 2026 Jul 6;32(38):e71351. doi: 10.1002/chem.71351 (PMC13660492; doi:10.1002/chem.71351)
Supplement: Supplementary file 1 [file CHEM-32-e71351-s001.pdf]

Supporting Information

Effects of Biotin on a Fluorescein-Based Photosensitizer Revealed by Multiscale Computational Modeling

Valeria Butera[a]+, Alessandra Gilda Ritacca[b]+, Giampaolo Barone[a], Nino Russo[b], Marta Erminia Alberto*[b]

[a] Dr V. Butera, Prof. G. Barone
Department of Biological, Chemical and Pharmaceutical Sciences (STeBiCeF), University of Palermo, Palermo, Italy

[b] Dr. A. G. Ritacca, Prof. N. Russo, Prof. M. E. Alberto,
Department of Chemistry and Chemical Technologies Department, University of Calabria, 87036 Arcavacata di Rende, CS, Italy

Pag.

Figure S1: MD Starting coordinates of the two systems containing one molecule of a) 1 and b) 1-biotin; **S1**

Figure S2: Replicas of the spontaneous insertion of 1 and 1-biotin within the POPC membrane; **S2**

Figure S3: MD Starting coordinates of the two systems containing five molecules per leaflet each of a) 1 and b) 1-biotin, without cholesterol and with cholesterol, c) and d), respectively; **S3**

Figure S4: Time-dependence of the Membrane Thickness (Å) and Area per lipid ($Å^2$) along with their average values without (Upper panels) and with cholesterol (Lower panels); **S4**

Figure S5. Radial distribution function, g(r) of a) 1 and 1-biotin; **S5**

Figure S6. Weighted distribution of hydrogen bond occupancies of 1 and 1-biotin; **S6**

Figure S7. Types of aggregates with different dimensions involving 1-biotin molecules; **S7**

Table S1. Relative perturbation effects upon the addition of 1 and 1-biotin molecules with and without cholesterol; **S8**

Figure S8: Computed UV-Vis spectra of 1, 1-biotin and their deprotonation forms **S10**

Figure S9: Molecular Orbital plots **S12**

Table S2: Energies (eV and nm), oscillator strength (*f*) and Experimental λ_{max} (nm) of the most relevant singlet-singlet transitions computed for all the protonation states of **1** and **1-biotin**; **S13**

Figure S10: Natural Transition Orbitals (NTOs) for the most relevant singlet-singlet transitions computed for all the protonation states of **1** and **1-biotin;** **S14**

Figure S11: Natural Transition Orbitals (NTOs) for the lowest three excited Triplet states, for all the protonation states of **1** and **1-biotin** **S15**

Table S3. Spin-orbit coupling matrix values (cm^{-1}) **S17**

Table S4. VEA and VIP Values (eV) in water, for compound **1-c**, **1-biotin**, computed at CAM-B3LYP, M11, wB97XD/6-31+G** level of theory. **S18**

Table S5. VEA and VIP Values (eV) in water, for compound **1**, **1-c**, **1-biotin** and **1-biotin**$^-$ in both singlet and triplet states and Thermodynamics of the Type I PDT reactions **S18**

Additional Computational Details **S20**

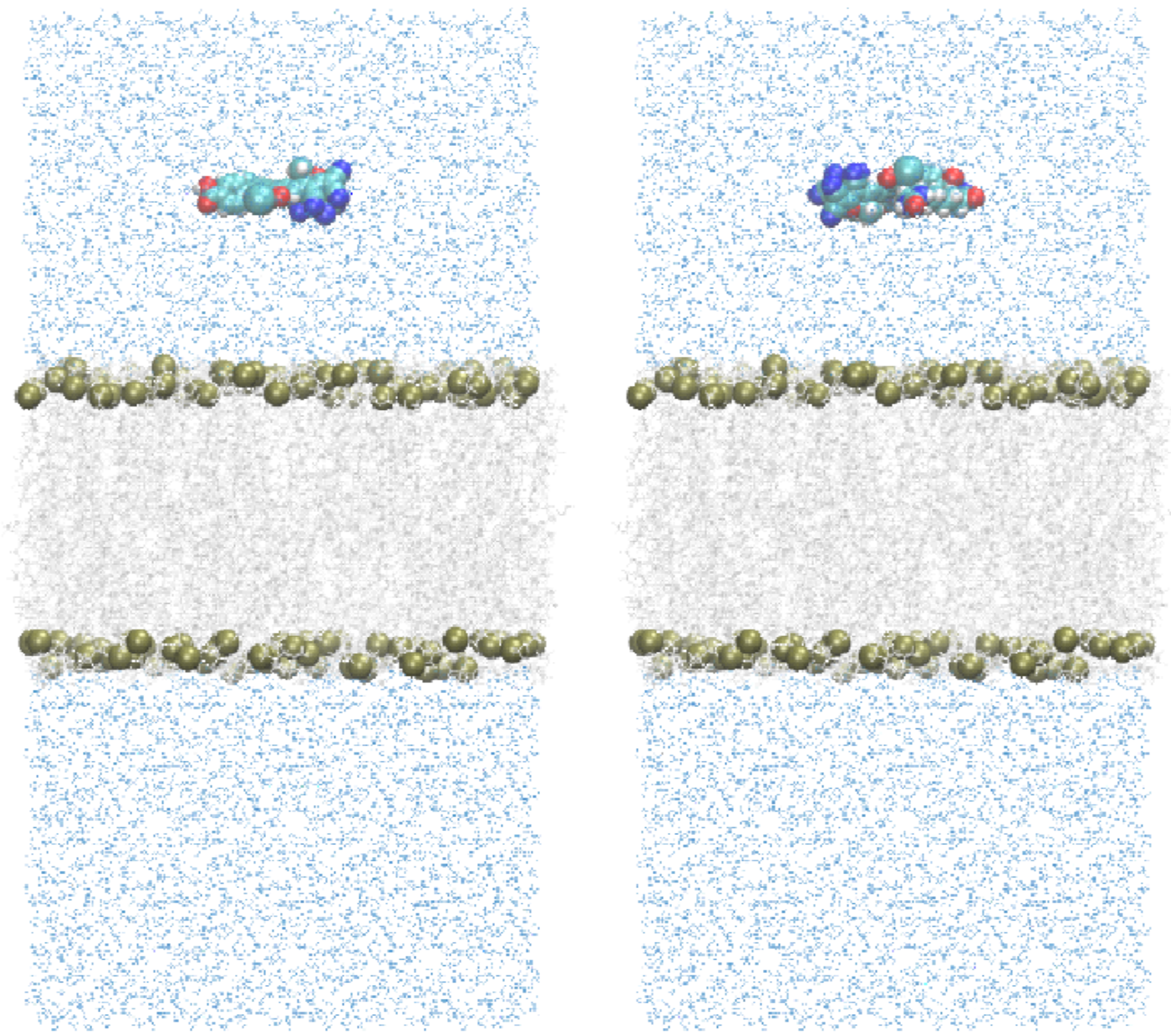

Figure S1. Starting coordinates of the two systems containing one molecule of a) **1** and b) **1-biotin,** in spheres, immersed in the bulk of water far from the bilayer slab. P atoms are depicted in tan spheres, lipids in grey lines and water in blue dots.

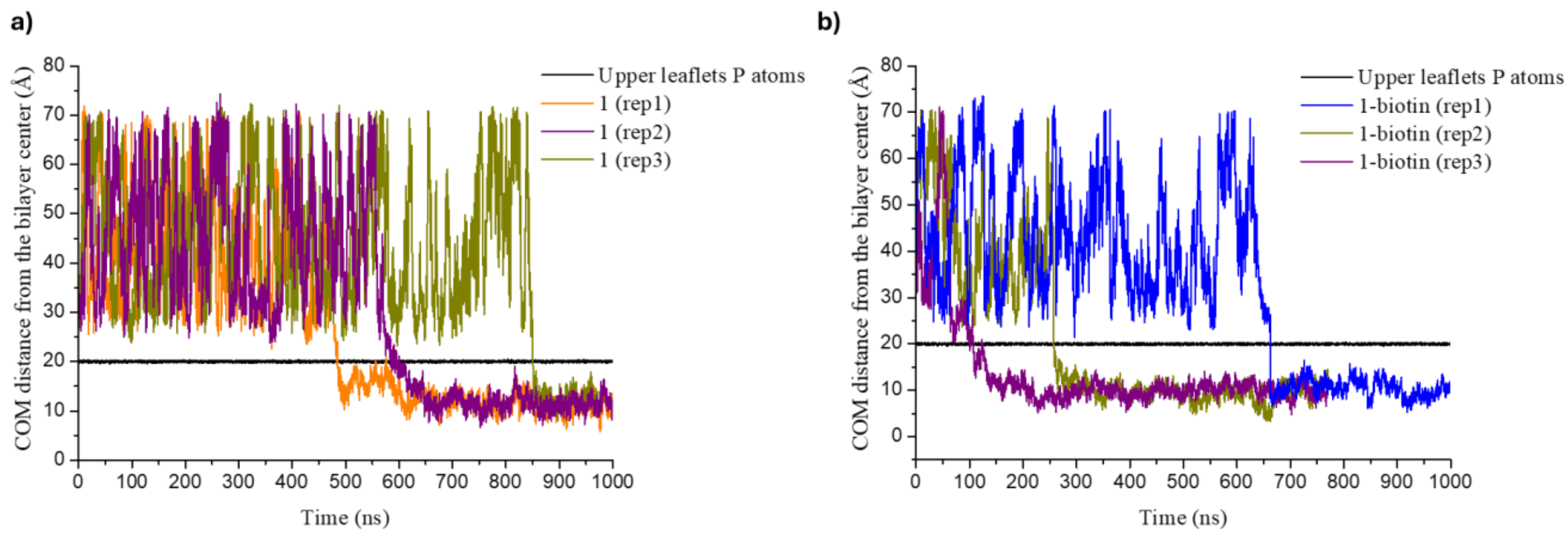

Figure S2: Time-dependence of the distance between the centre of mass (COM) of a) **1** and b) **1-biotin** and the bilayer centre, in the three independent replica simulations performed for each system (rep 1-3). The black line is the centre of mass of P atoms of the upper layer of the membrane.

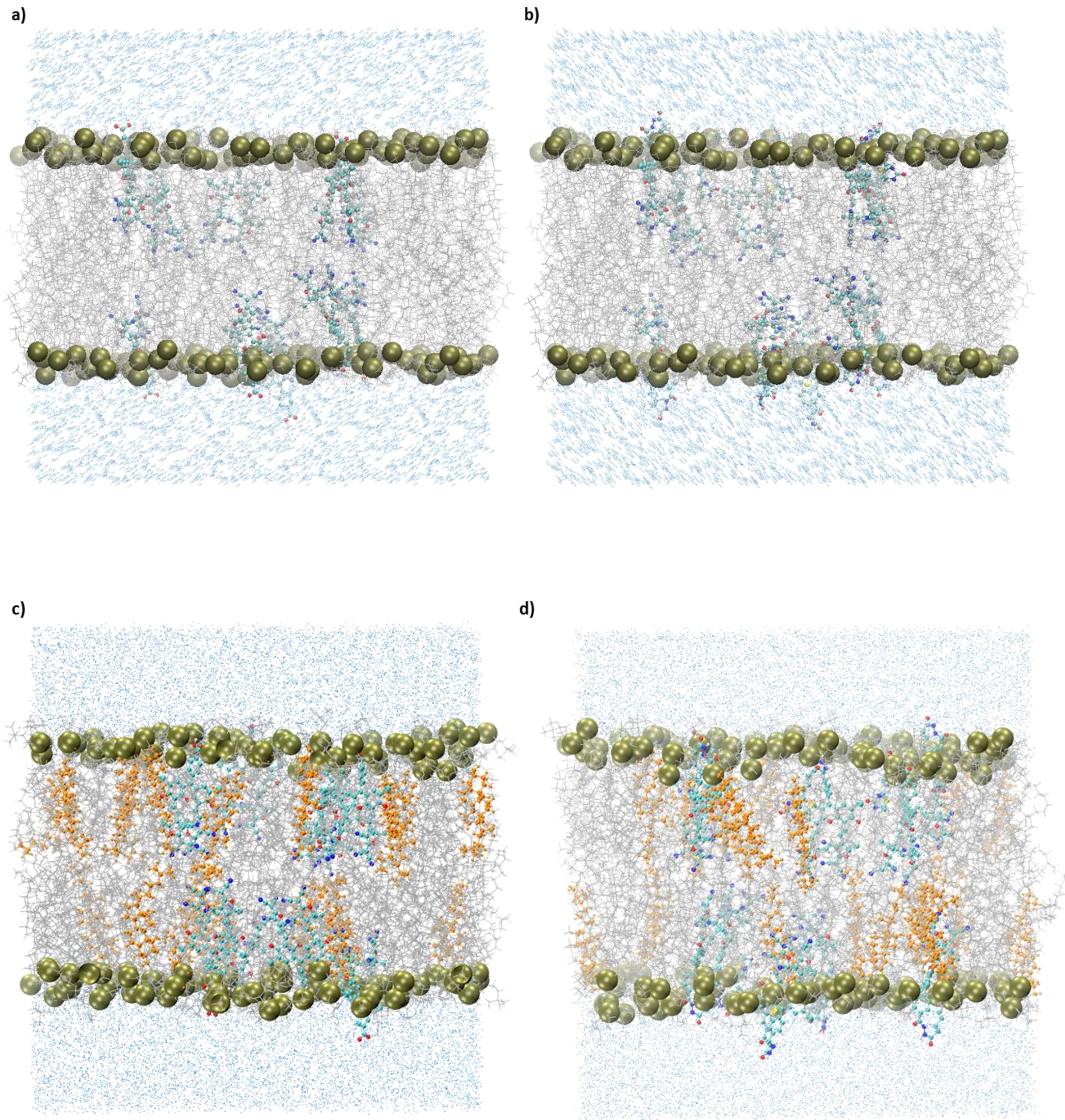

Figure S3. Starting coordinates of the systems containing five molecules per leaflet each of a) **1** and b) **1-biotin,** in cpk, already embedded in a pure POPC membrane and c) **1** and d) **1-biotin**, in cpk, embedded in a mix of POPC/CHL membrane, with cholesterol molecules in cpk. P atoms are depicted in tan spheres, lipids in grey lines, CHL are orange and water in blue dots.

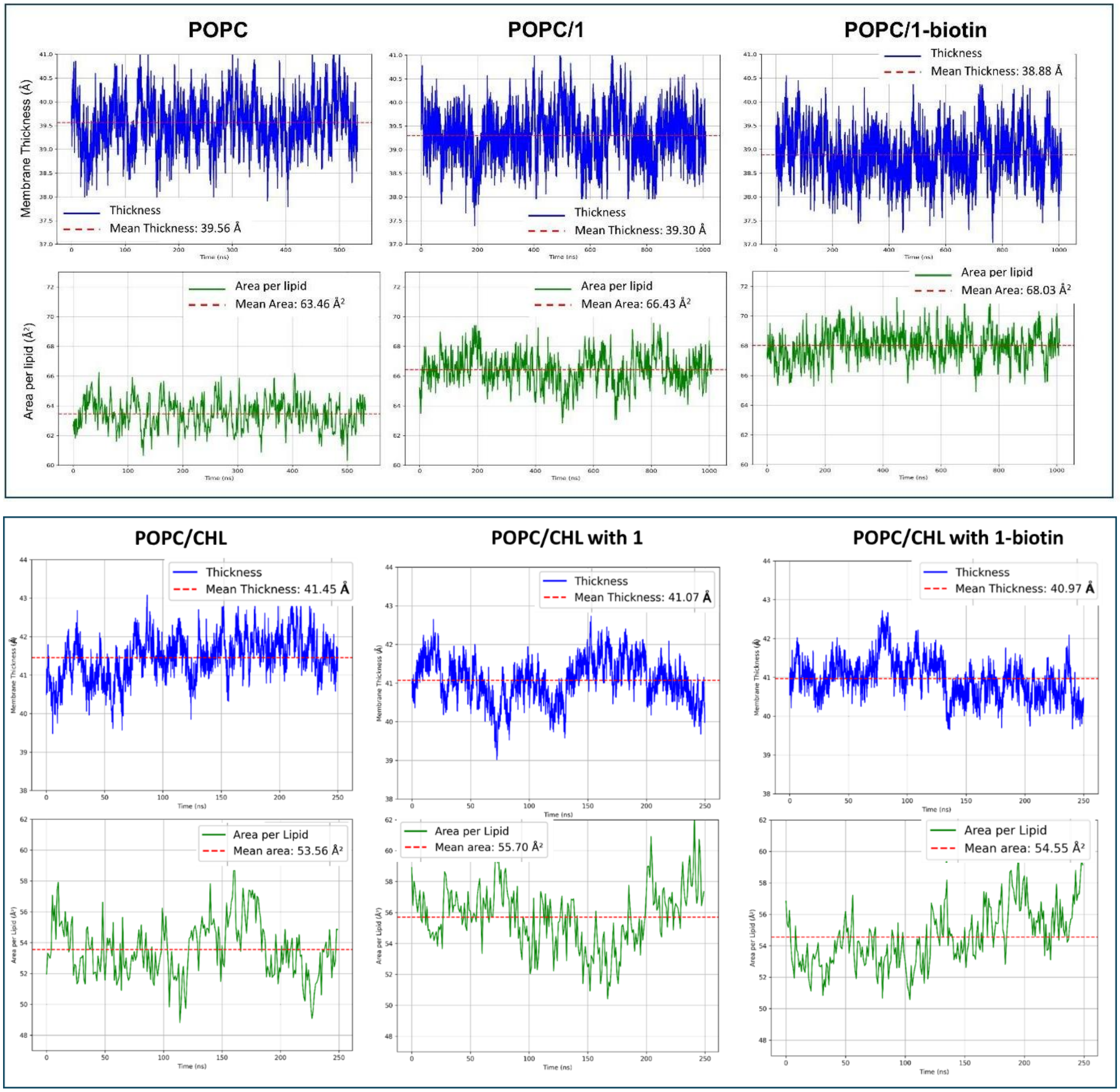

Figure S4: Time-dependence of the Membrane Thickness (Å) (blue lines) and Area per lipid (Å^2) (green lines) extracted from (Upper panels) the 1μs trajectories of the MD simulations of **POPC** membrane and the systems containing five molecules per leaflet each of 1 (**POPC/1**) and 1-biotin (**POPC/1-biotin**), and (Lower panels) from 250 ns of trajectories of the MD simulations of **POPC/CHL** membrane, and the systems containing five molecules per leaflet each of 1 (**POPC/CHL/1**) and 1-biotin (**POPC/CHL/1-biotin**).

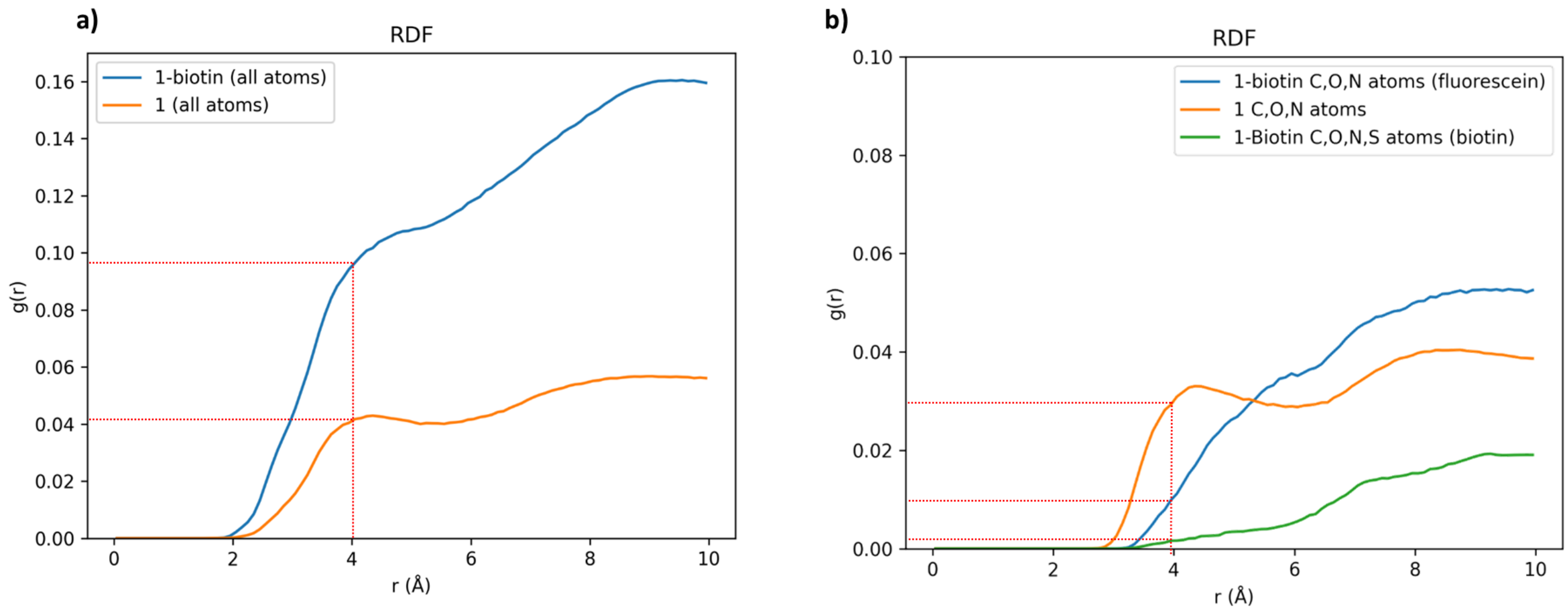

Figure S5. Radial distribution function, g(r) of a) **1** and **1-biotin**, calculating the number of molecules found as a function of distance r(Å), normalized by the expected number of molecules at that distance and b) fluorescein and biotin fragments belonging to **1** and **1-biotin**, without H-atoms. a) Blue (**1-biotin**) and orange (**1**) lines, show the probability to find all the atoms of the species at a certain distance (4 Å). b) The green (**1-biotin**) line indicates the probability to find only C,O,N and S atoms of the biotin fragment, while blue (**1-biotin**) and orange (**1**) refer to C, O and N atoms of the fluorescein part of the two photosensitizers. The dotted red lines indicate the higher probability to find **1** and **1-biotin** aggregates at the desired distance (4Å).

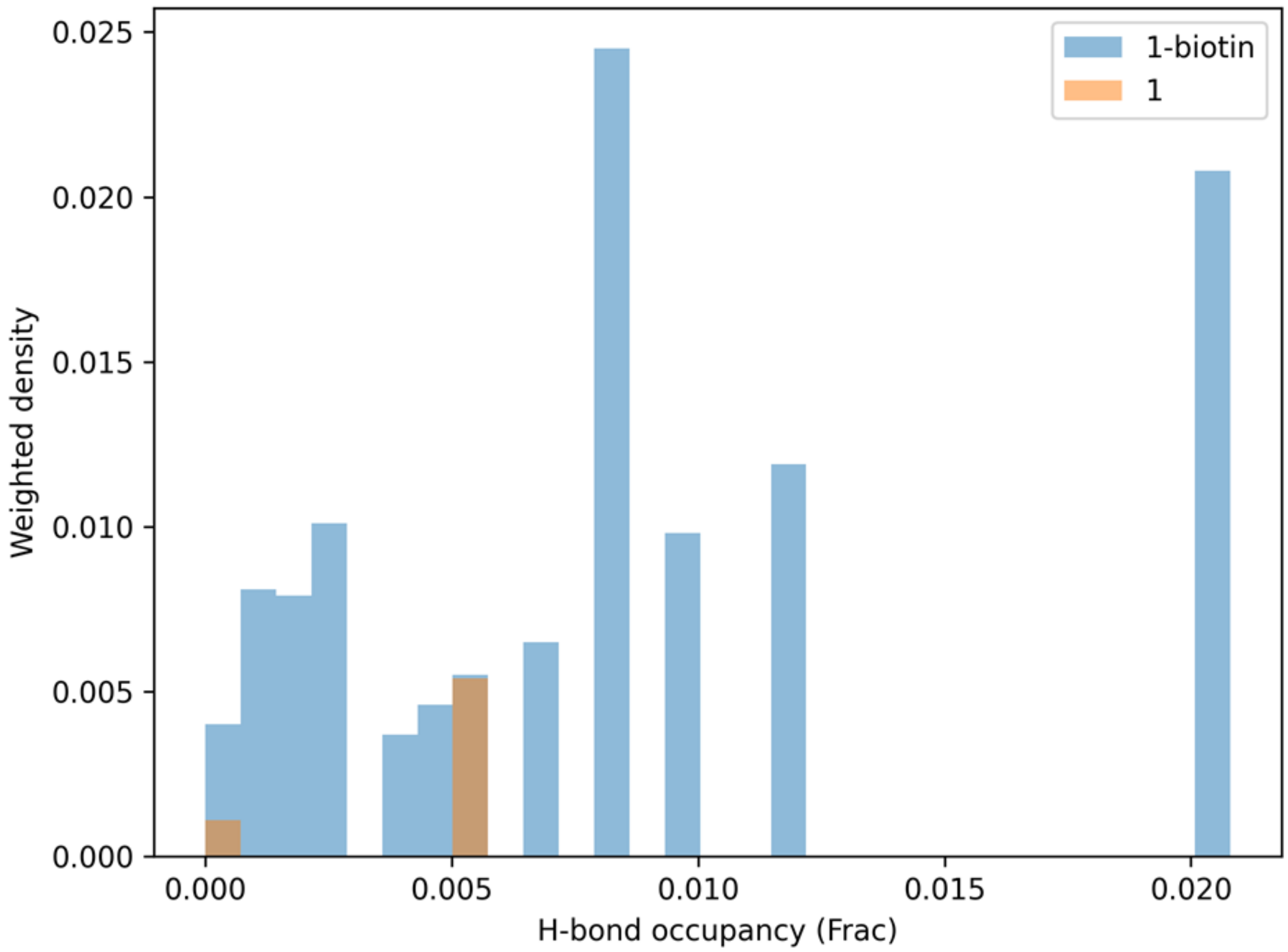

Figure S6. Weighted distribution of hydrogen bond occupancies (Frac) of **1** (orange) and **1-biotin** (blue), derived by H-bond analysis performed with CPPTRAJ tool. Each interaction is weighted by its occupancy to emphasize persistent hydrogen bonds.

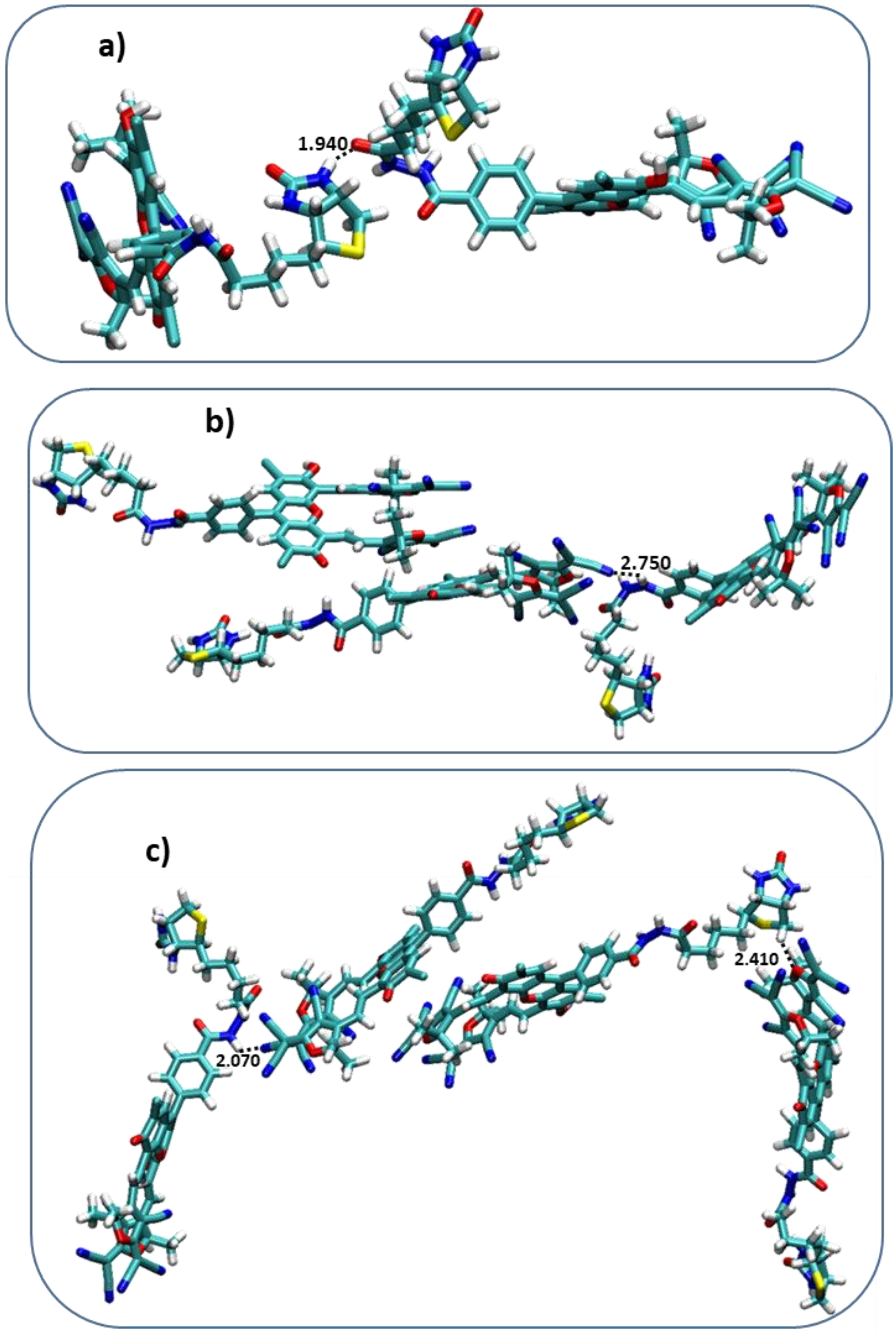

Figure S7. Types of aggregates, with different dimensions, that occur during the simulation involving **1-biotin** molecules: a) two, b) three and c) four. The reported most frequent h-bond lengths are in Å.

Table S1. Structural properties of POPC membrane in presence and in absence of ca. 11% of cholesterol and the relative perturbation effects upon the addition of **1** and **1-biotin** molecules. The values are reported in Å^2 and Å for area per lipid and membrane thickness respectively.

MD systems	Area per lipid (Å^2)	Membrane thickness (Å)
POPC	63.46	39.56
POPC/CHL	53.56	41.45
POPC/1	66.43	39.30
POPC/CHL/1	55.70	41.07
POPC/1-biotin	68.03	38.88
POPC/CHL/1-biotin	54.55	40.97

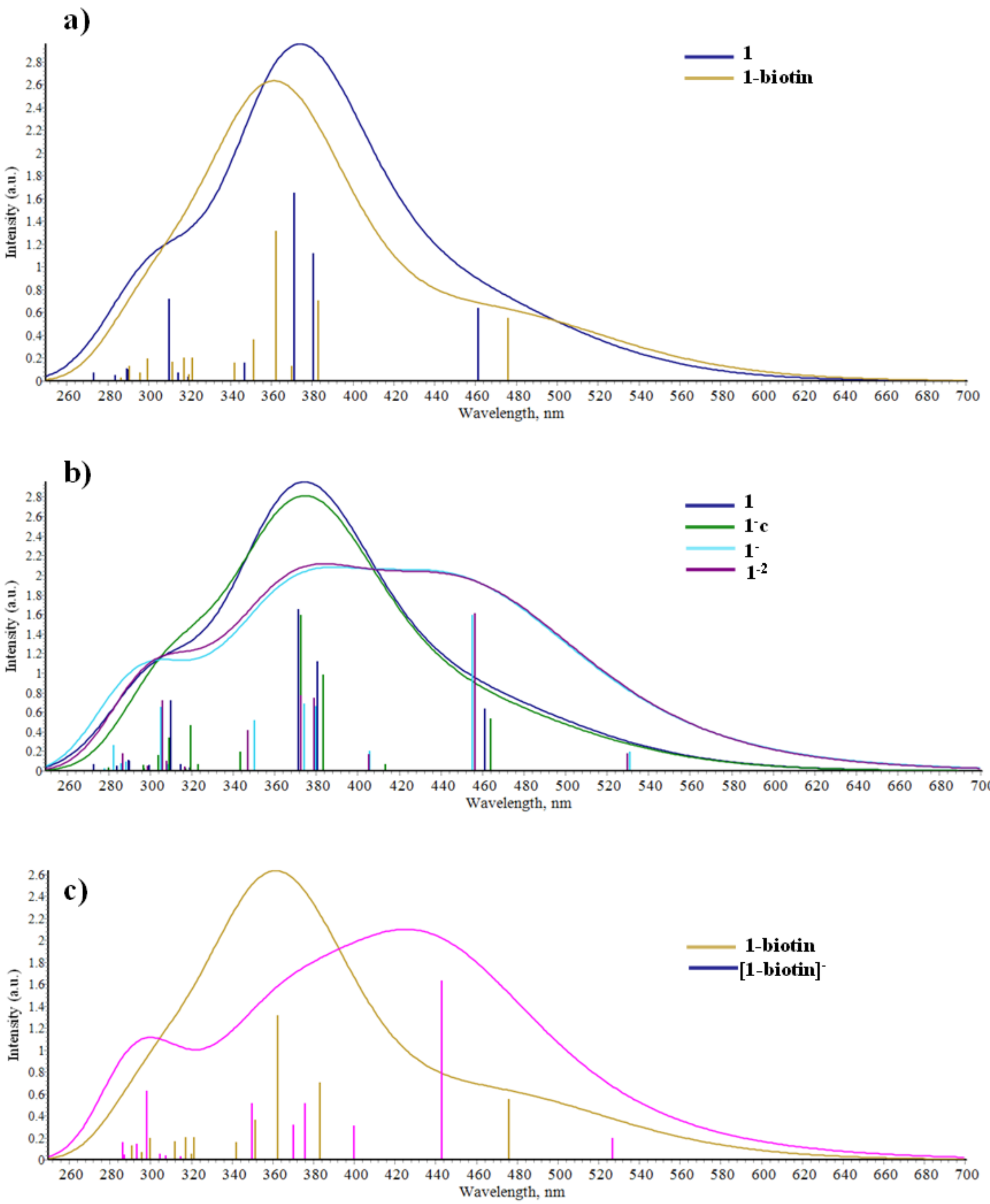

a)
1
1-biotin
Intensity (a.u.)
Wavelength, nm
b)
1
1⁻c
1⁻
1⁻²
Intensity (a.u.)
Wavelength, nm
c)
1-biotin
[1-biotin]⁻
Intensity (a.u.)
Wavelength, nm

d)

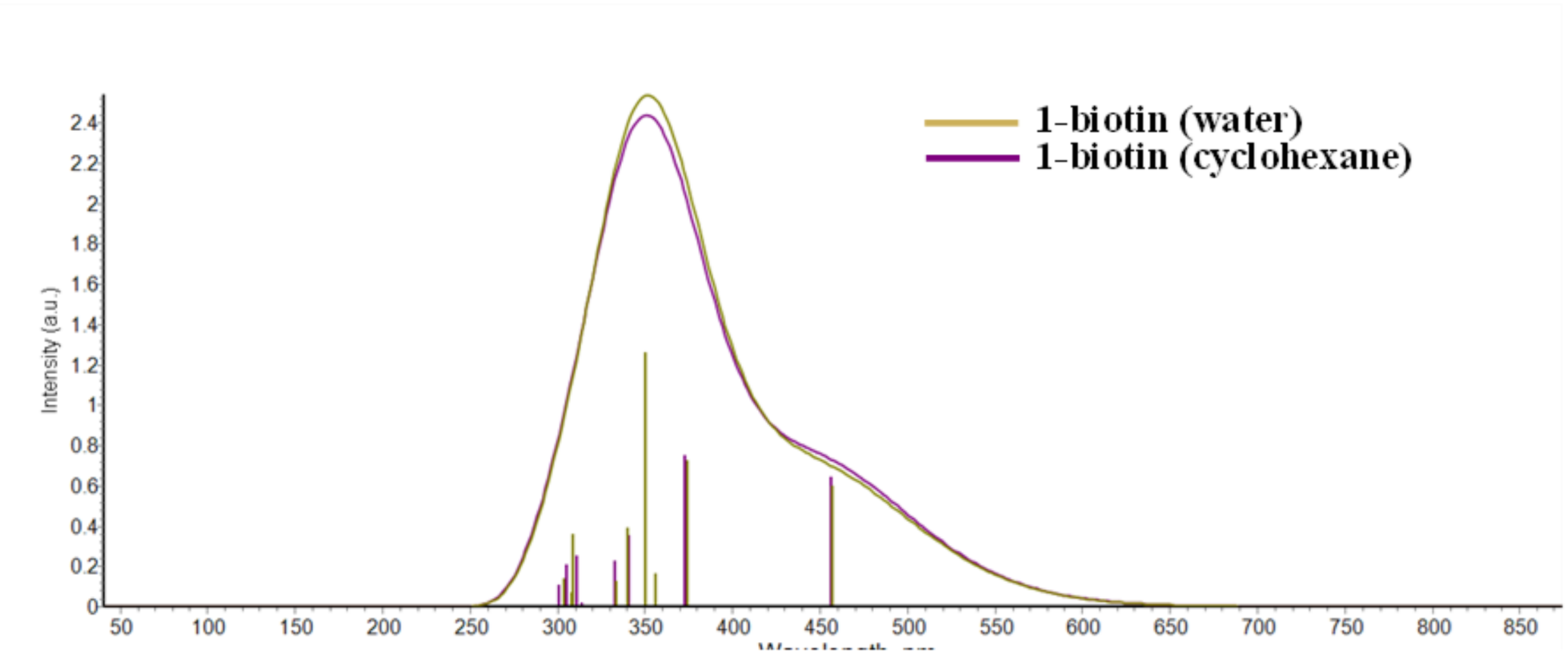

e)

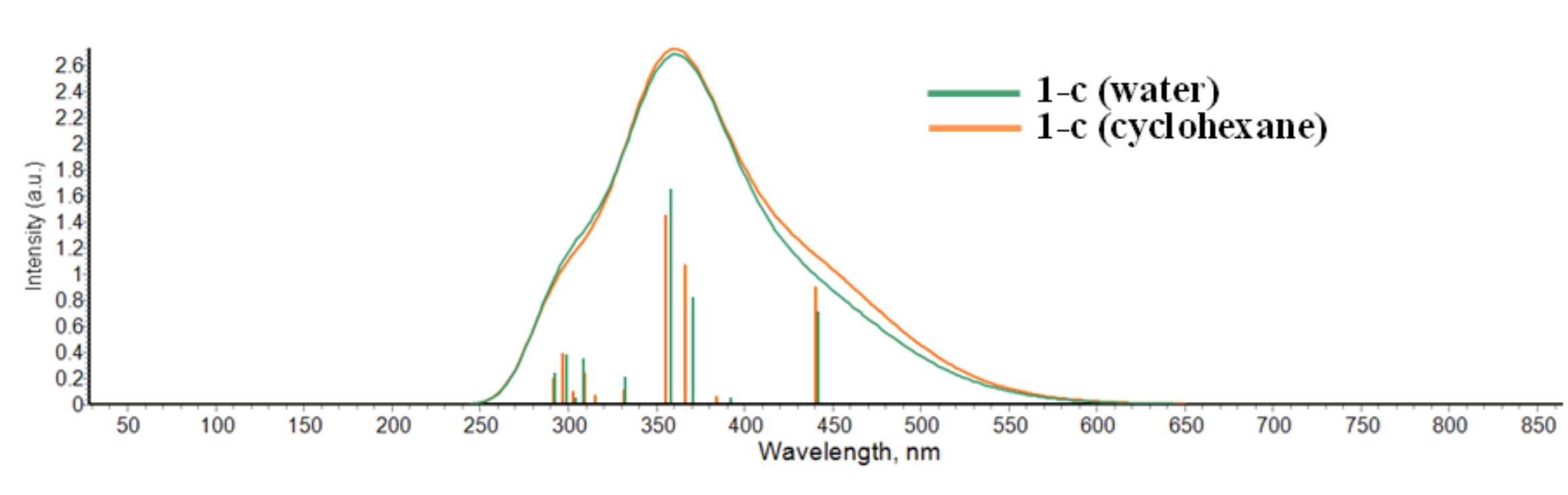

Figure S8: a-c) Computed UV-Vis spectra of **1**, **1-biotin** and their deprotonation forms, in water; superimposition of UV-Vis spectra of d) **1-biotin** and e) **1-c**, computed in water and cyclohexane

Table S2: Energies (eV and nm), oscillator strength (*f*) and Experimental λ_{max} (nm) of the most relevant singlet-singlet transitions computed for all the protonation states of **1** and **1-biotin**, at the TDDFT/CAM-B3LYP/6-31+G(d,p) level of theory, in water environment.

**Compound**		**ΔE (eV)**	**λ (nm)**	**f**	**λ_{exp}(nm)[8]**
**1**	S1	2.69	461	0.637	≈ 490
	S3	3.26	381	1.121	
	S4	3.34	371	1.650	
	S5	3.57	347	0.161	
**1^{-}c**	S1	2.67	464	0.538	
	S3	3.23	383	0.980	
	S4	3.33	373	1.596	
	S5	3.61	343	0.195	
**1^{-2}**	S1	2.34	530	0.179	>600
	S2	2.71	457	1.607	
	S3	3.06	405	0.172	
	S4	3.27	379	0.747	
	S5	3.23	373	0.771	
	S6	3.57	347	0.417	
**1-biotin**	S1	2.60	476	0.554	≈ 490
	S2	2.24	383	0.703	
	S3	3.35	370	0.131	
	S4	3.42	362	1.314	
	S5	3.53	352	0.365	
**[1-biotin]$^{-}$**	S1	2.35	527	0.195	
	S2	2.80	443	1.627	
	S3	3.10	400	0.307	
	S4	3.30	376	0.514	
	S5	3.35	370	0.320	
	S6	3.54	350	0.516	

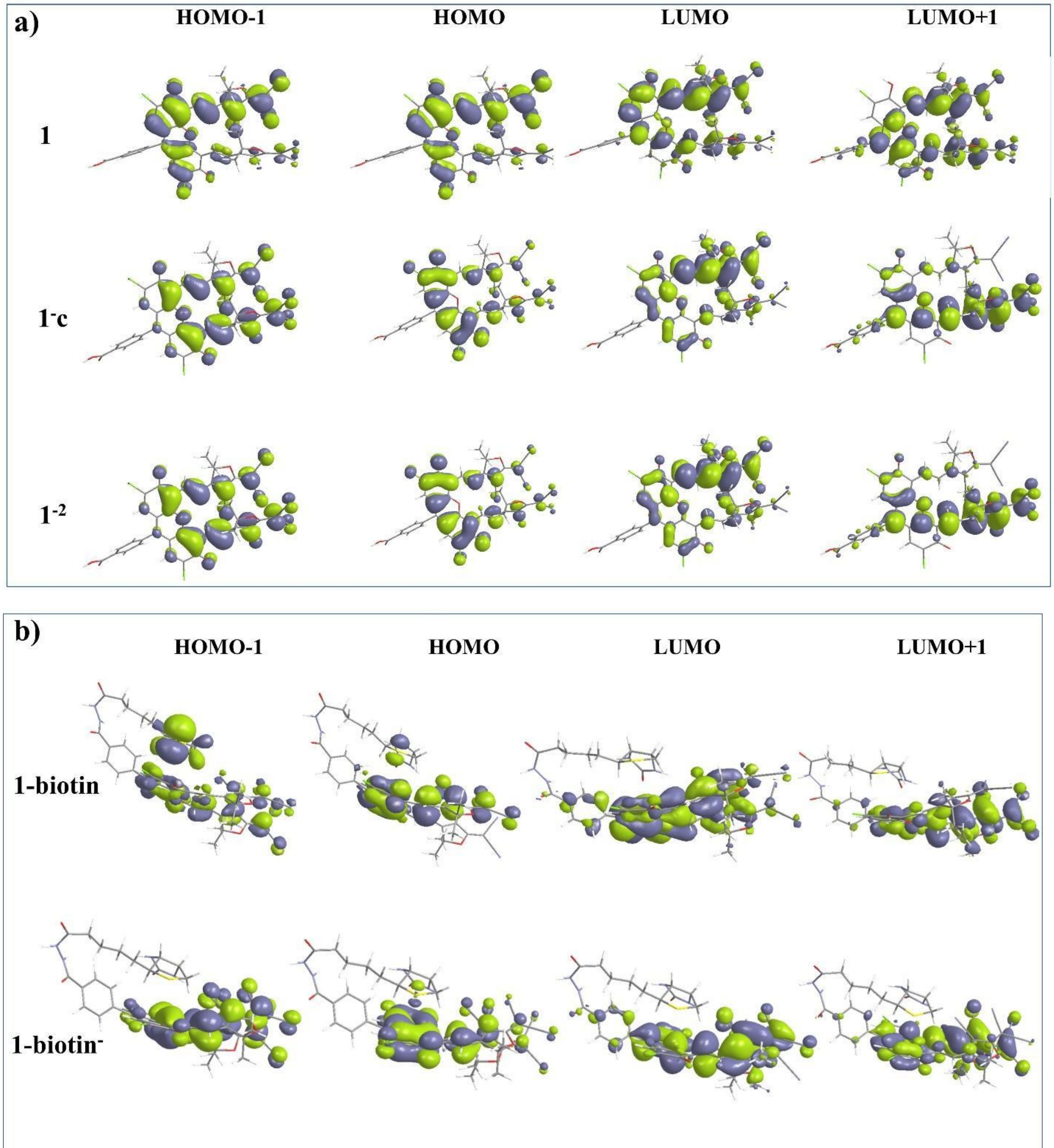

Figure S9: HOMO-1, HOMO, LUMO and LUMO+1 Molecular Orbital's plots for **a)** compound **1** and its two deprotonated species $\mathbf{1^{-}_{c}}$ and $\mathbf{1^{-2}}$; **b) 1-biotin** and its anionic form **1-biotin**$^{-}$, at TD-CAM-B3LYP/6-31+G(d,p)/water/PCM level of theory.

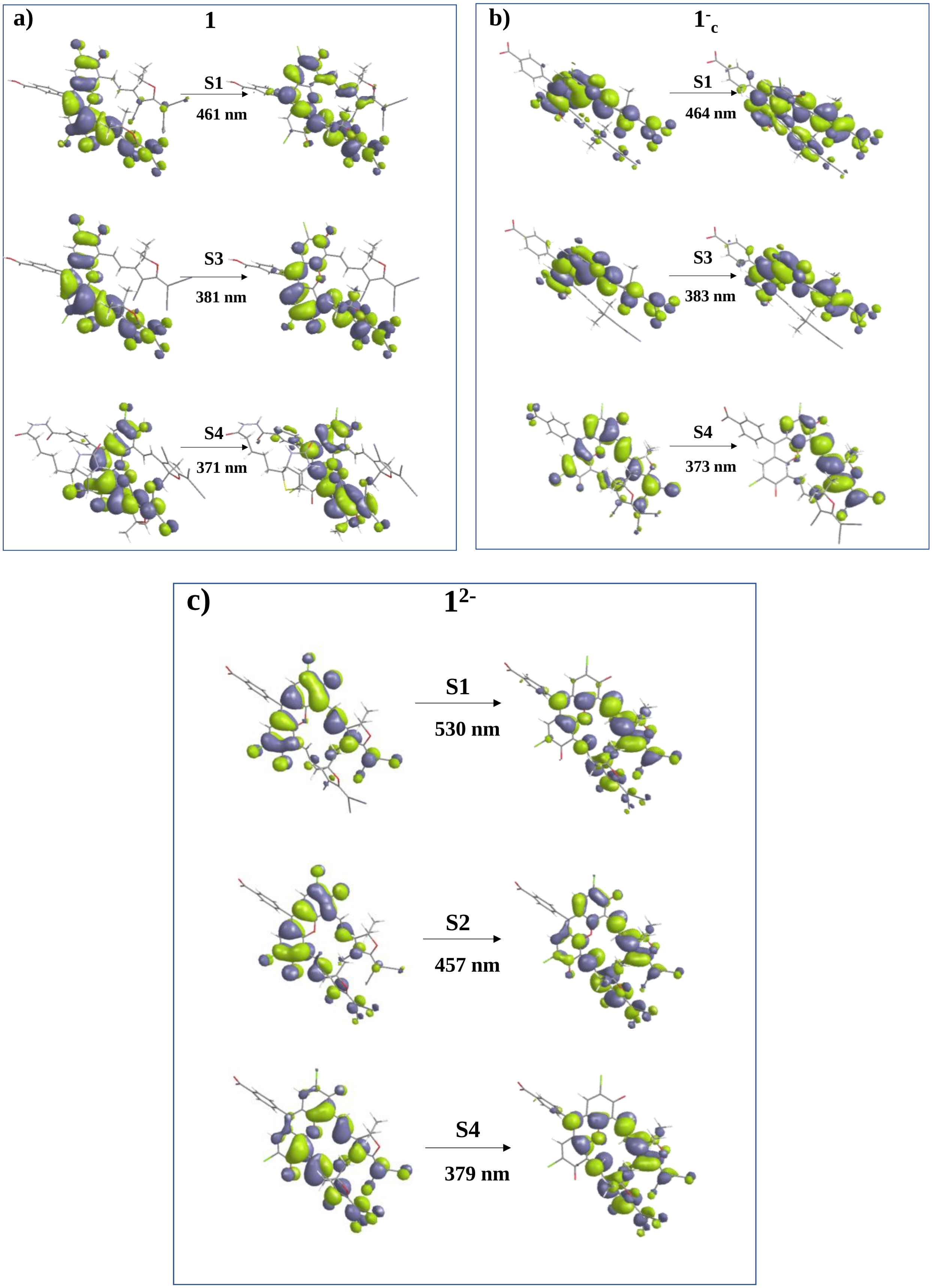

a)
1
S1
461 nm
S3
381 nm
S4
371 nm
b)
1⁻c
S1
464 nm
S3
383 nm
S4
373 nm
c)
1²⁻
S1
530 nm
S2
457 nm
S4
379 nm

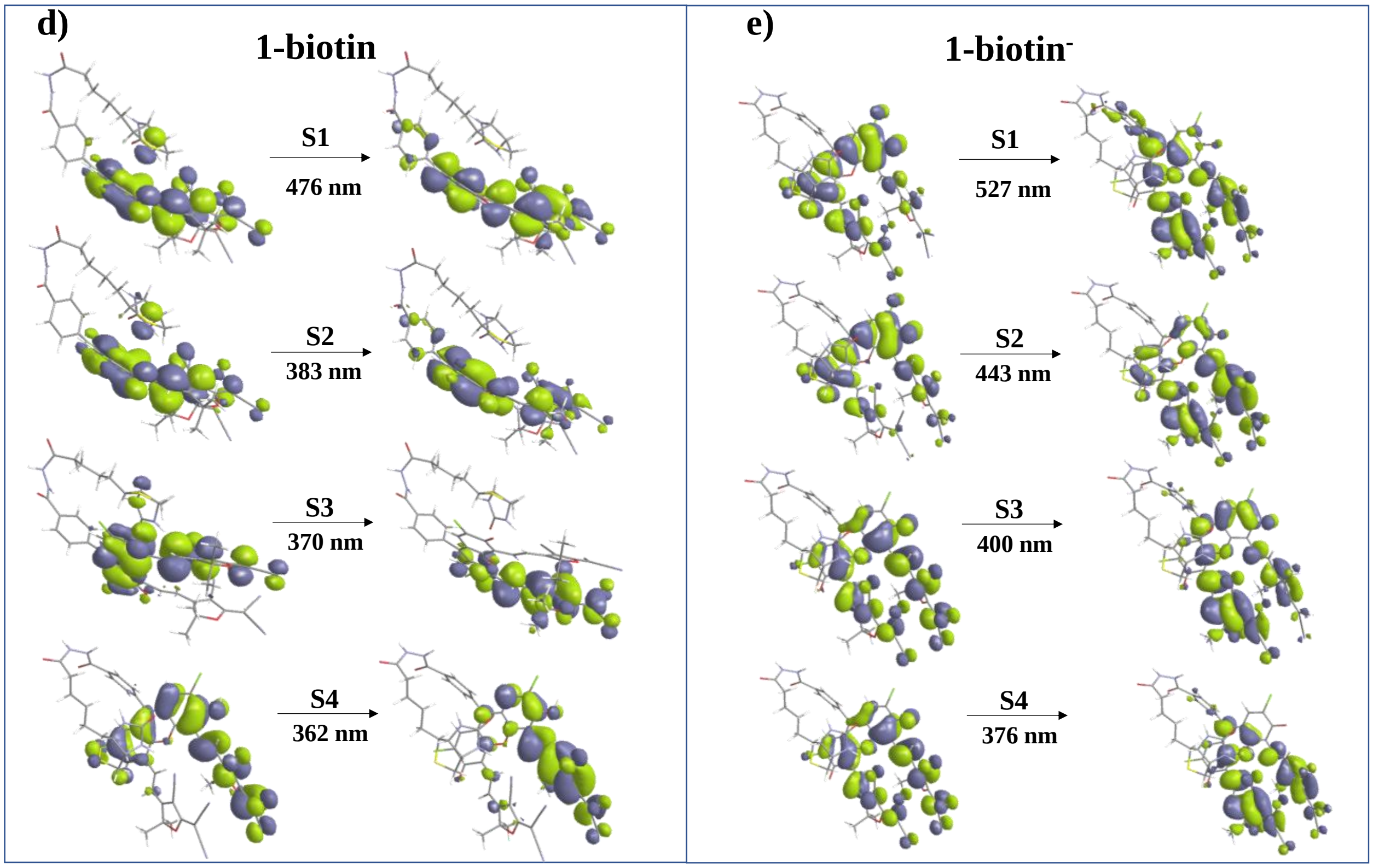

Figure S10: Natural Transition Orbitals (NTOs) that describe the main singlet-singlet transitions computed for all the protonation states of **1** and **1-biotin (a-e)**, at the TDDFT/CAM-B3LYP//6-31+G(d,p)/water/PCM level of theory, in water environment.

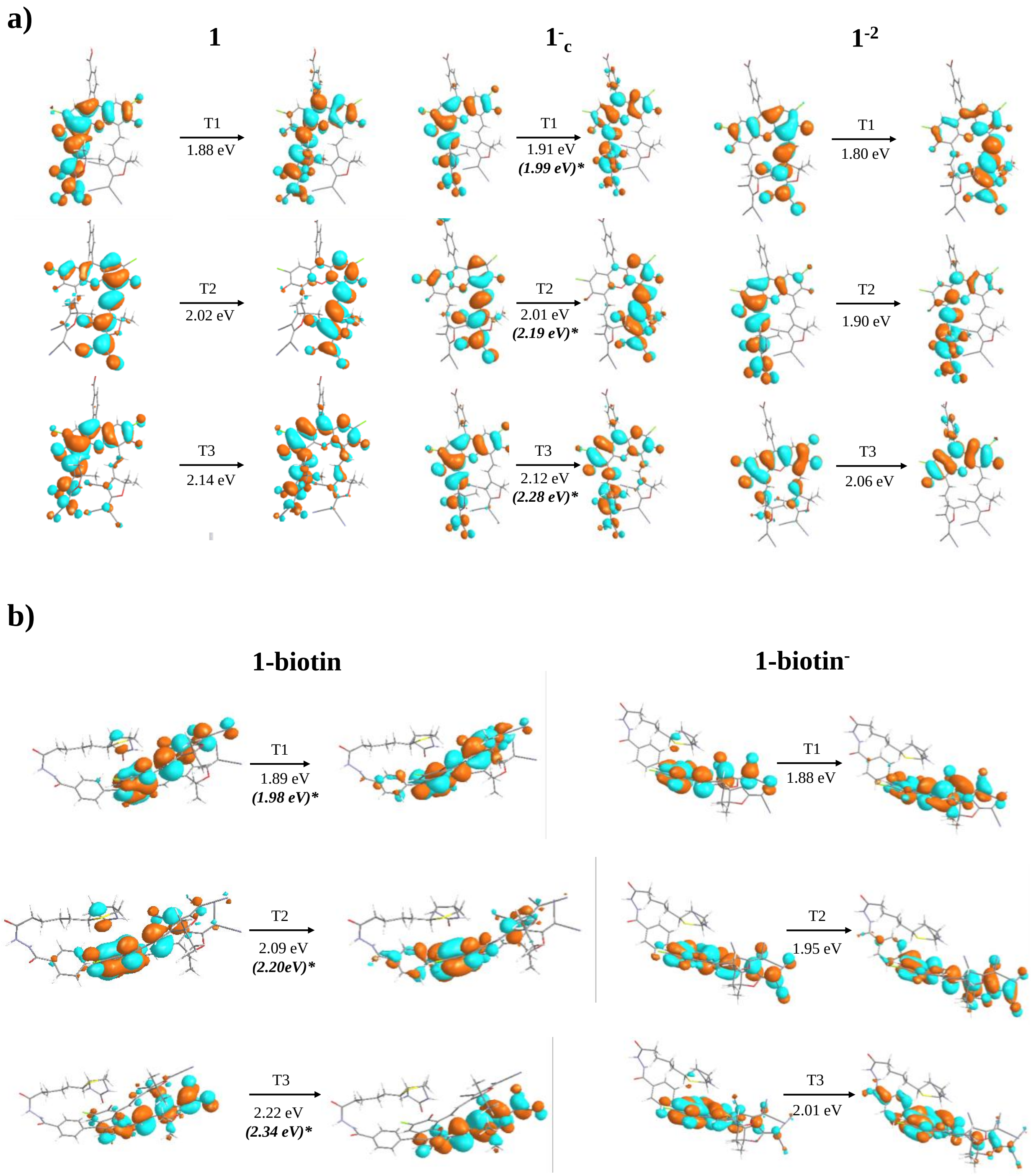

Figure S11: Natural Transition Orbitals (NTOs) that describe the lowest three triplet excited states (T_1-T_3) for **a)** compound **1** and its two deprotonated species $\mathbf{1^{-}_{c}}$ and $\mathbf{1^{-2}}$; **b) 1-biotin** and its anionic form $\mathbf{1\text{-}biotin^{-}}$, at TD-CAM-B3LYP/6-31+G(d,p)/water/PCM level of theory. For 1-c and 1-biotin, T_1-T_3 energies in cyclohexane are also reported in parenthesis *()**

Table S3. Spin-orbit coupling matrix values (cm^{-1}) calculated between the lowest singlet excited state S_1 and the three lowest triplet states below it, for **1^-c** and **1-biotin**, in water and cyclohexane

**Water**	**1^-_c**		**1-biotin**	
**S_1-T_m**	**SOC(S_1-T_m)**	**ΔE(S_1-T_m)**	**SOC(S_1-T_m)**	**ΔE(S_1-T_m)**
**S_1-T_1**	0.20	0.75	0.15	0.71
**S_1-T_2**	0.16	0.66	0.48	0.51
**S_1-T_3**	0.40	0.55	0.45	0.39

**Cyclohexane**	**1^-_c**		**1-biotin**	
**S_1-T_m**	**SOC(S_1-T_m)**	**ΔE(S_1-T_m)**	**SOC(S_1-T_m)**	**ΔE(S_1-T_m)**
**S_1-T_1**	0.17	0.82	0.05	0.74
**S_1-T_2**	0.83	0.62	0.57	0.52
**S_1-T_3**	0.54	0.53	0.2	0.38

Table S4: VEA and VIP Values (eV) in water, for compound **1-c** and **1-biotin**, computed at CAM-B3LYP, M11, wB97XD/6-31+G** level of theory.

	**Cam-B3LYP**			
	VEA (S0)	VIP (S0)	VEA(T1)	VIP(T1)
**1biotin**	-3.62	6.53	-5.52	4.64
**1-c**	-3.97	6.19	-5.89	-3.97
**O_2**	-3.67			
	**M11**			
	VEA (S0)	VEA (S0)	VEA (S0)	VEA (S0)
**1biotin**	-3.77	-3.77	-3.77	-3.77
**1-c**	-3.90	-3.90	-3.90	-3.90
**O_2**	-3.80	-3.80	-3.80	-3.80
	**wB97XD**			
**1biotin**	-3.67	6.27	-5.72	4.22
**1-c**	-3.79	6.20	-5.86	4.13
**O_2**	-3.55			

Table S5. a) VEA and VIP Values (eV) in water, for compound 1, 1-biotin and their anionic forms, in both singlet and triplet states. All values were computed at CAM-B3LYP/6-31+G** level of theory, including GD3 dispersion forces. The calculated value for the VEA of the $^2O_3$ at the same level of theory is -3.69 eV; b) Thermodynamics [ΔE= E(products) –E(reactants)] of the Type I PDT reactions based on the VEA and VIP values reported in a).

a)	VIP	VEA	VIP(T1)	VEA(T1)
**1**	6.24	-3.88	4.36	-5.76
**1^-_c**	6.19	-3.97	4.28	-5.89
**1^{2-}**	5.65	-3.57	3.85	-5.37
**1-biotin**	6.53	-3.62	4.64	-5.52
**1-biotin$^-$**	5.62	-3.55	3.95	-5.22

b)	Type I Photoreactions	1	1^-c	1^{2-}	1-biotin	1-biotin$^-$
	**Autoionization Reactions**					
(1)	$^3Ps + {}^1Ps \rightarrow {}^{\bullet}Ps^+ + {}^{\bullet}Ps^-$	0.48	0.30	0.28	1.01	0.4
(2)	$^3Ps + {}^3Ps \rightarrow {}^{\bullet}Ps^+ + {}^{\bullet}Ps^-$	-1.4	-1.61	-1.52	-0.88	-1.27
	**Indirect Reaction**					
(3)	${}^{\bullet}Ps^- + {}^3O_2 \rightarrow {}^1Ps + {}^{\bullet}O_2^-$	0.19	0.28	-0.12	-0.07	-0.14
	**Direct Electron transfer**					
(4)	$^1Ps + {}^3O_2 \rightarrow {}^{\bullet}Ps^+ + {}^{\bullet}O_2^-$	2.55	2.50	1.96	2.84	1.93
(5)	$^3Ps + {}^3O_2 \rightarrow {}^{\bullet}Ps^+ + {}^{\bullet}O_2^-$	0.67	0.59	0.16	0.95	0.26

Additional Computational details -pKa calculation-

The values of the pK_a were calculated following the computational methodology developed by Galano and collaborators.[1] The authors used a fitting parameters (FP) method to derive two empirically fitted parameters, m and C_0, for three different class of chemical compounds, namely phenols, carboxylic acids, and amines, at 74 levels of theory. The standard linear regression fit necessary to obtain m and C_0 empirical parameters was obtained using a training set of 20 molecules for each chemical family, according to:

$$pK_a^{exp} = m\Delta G_{s(BA)} - C_0$$

where $\Delta G_{s(BA)}$ represents the difference in Gibbs free energy between each base (B) and its corresponding conjugated acid (A) in water solvent. The obtained parameters were then employed to test the performance of the FP method using a set of ten molecules for each chemical family.

Those parameters have been shown to allow for the prediction of reliable pK_a^{calc} values by calculating only the Gibbs energy difference between an acid and its conjugated base in aqueous solution. The pK_a^{calc} values calculated with the FP method show good agreement with the experimental data, with values of mean unsigned errors (MUE), maximum absolute errors (MAE), and root-mean square errors (RMSE) that are below 0.5 pK_a units, 1.0 pK_a units and 0.5 pK_a units, respectively, thus being significantly below the 2 pK_a units currently accepted as reasonably accurate for pK_a calculations.

The same authors provided also a methodology to quantify the population of each acid-base form by calculating their molar fractions (f)[2]. For any generic acid, the molar fraction of the fully deprotonated species can be calculated as:

$$f(X^{n-}) = \frac{1}{1 + \sum_{i=1}^{n} \beta_i [H^+]^i}$$

While that of any acid-base species is obtained from:

$$f\left(H_i X^{(n-1)}\right) = \beta_i [H^+]^i f(X^{n-})$$

Where

$$\beta_i = 10^{\sum_{j=1}^{i} pKa_{(n+1-j)}}$$

[1] J. Chem. Inf. Model. 2016, 56, 1714–1724. DOI: 10.1021/acs.jcim.6b00310
[2] https://onlinelibrary.wiley.com/doi/epdf/10.1002/jcc.23409
